# Increased expression of NAF1 contributes to malignant phenotypes of glioma cells through promoting protein synthesis and associates with poor patient survival

**DOI:** 10.1038/s41389-019-0134-2

**Published:** 2019-04-01

**Authors:** Jing Wei, Qi Yang, Jing Shi, Bingyin Shi, Meiju Ji, Peng Hou

**Affiliations:** 1 Key Laboratory for Tumor Precision Medicine of Shaanxi Province and Department of Endocrinology, The First Affiliated Hospital of Xi’an Jiaotong University, Xi’an, 710061 China; 2 Center for Translational Medicine, The First Affiliated Hospital of Xi’an Jiaotong University, Xi’an, 710061 China

## Abstract

The H/ACA ribonucleoprotein (RNP) complex noncore subunit NAF1 is an indispensable factor during H/ACA RNP maturation, and one of the widely known functions of H/ACA RNP is modulating ribosome biosynthesis. However, the specific biological role and exact mechanism of NAF1 in human cancers including glioma remain largely unclear. In this study, we found that NAF1 was highly expressed in gliomas relative to normal brain tissues, and demonstrated that increased expression of NAF1 was strongly correlated with poor patient survival. Further studies revealed that NAF1 was transcriptionally regulated by c-Myc, NRF2, and telomerase reverse transcriptase (TERT), which are the key molecules associated with malignant progression of gliomas. Moreover, we demonstrated that NAF1 was a functional oncogene in glioma cells through promoting cell growth in vitro and in vivo, survival, migration, and invasion. Mechanistically, NAF1 acted as a rate-limiting controller of cell growth and invasiveness through enhancing 40S subunit assembly and protein synthesis including c-Myc, NRF2, TERT, POLR1A, and POLR2A. These molecules in turn enhanced the transcription and translation of NAF1, thereby forming positive feedback loops between them to promote malignant phenotypes of glioma cells. In addition, our data also showed that NAF1 depletion could trigger ribosome stress, not only impairing ribosomal biosynthesis but also reactivating p53 signaling via blocking MDM2. Taken together, we demonstrated that NAF1 promotes the tumorigenesis and progression of glioma through modulating ribosome assembly and protein synthesis, and predicted that NAF1 may be a potential therapeutic target and valuable prognostic biomarker in gliomas.

## Introduction

Gliomas, which account for about 2–4% of all systemic malignant tumors, comprise about 30% of all brain tumors and central nervous system tumors, and 75% of all malignant brain tumors^1^. In general, the incidence rate of gliomas is about six per type of brain tumor 100,000 annually, and 17,000 new cases of glioblastoma multiforme are diagnosed per year^2^. Although with significant progress in comprehensive therapy, the 5-year prognosis of glioma patients is still poor^3,4^. Thus, it is pressing to clearly illustrate the mechanism of glioma tumorigenesis and identify more valuable prognostic biomarkers and effective therapeutic targets for this disease.

Ribosome biogenesis and protein synthesis play indispensable roles during normal cell growth as well as in tumorigenesis^5,6^. Increased tumor susceptibility is associated with changes in ribosomal activity, which may be caused by speeded protein synthesis rate and increased translation of specific cancer-related mRNAs^7,8^. Mutations in ribosome biogenesis are connected to several human ribosomal genetic diseases, including inherited bone marrow failure syndromes^9^. In addition, the dysregulation of ribosomal may also be linked to muscle wasting^10^.

The mammalian H/ACA box ribonucleoproteins (RNPs) consist of only one of the 100–200 diverse box H/ACA small nucleolar RNA (snoRNA) and the evolutionary conserved four key proteins including NAP57, NOP10, NHP2 (forming the core trimer), and GAR1. They perform several basic biological functions, such as protein synthesis, gene expression, and chromosome stability^9,11^. NAF1, an H/ACA box RNP assembly chaperone, is a factor necessary for H/ACA snoRNP maturation and ribosome biosynthesis in mammalian cells^12^. In addition, among these RNA biogenesis factors, NAF1 may be unique in that it is required at full dosage for telomerase and telomere maintenance in the process of pulmonary fibrosis–emphysema^13^. Moreover, there is also evidence showing that single nucleotide polymorphisms (SNPs) in NAF1 are associated with cancer risk probably through affecting telomere length^14,15^; however, to our knowledge, no studies are available to define its role in human cancers, especially in gliomas.

In the present study, we observe that NAF1 is dramatically upregulated in gliomas, and find a close connection between increased expression of NAF1 and poor patient survival. Moreover, by a series of in vitro and in vivo experiments, we demonstrate that NAF1 may be a potent oncogene in glioma cells. Further studies reveal that NAF1 promotes glioma tumorigenesis and progression through enhancing ribosome biogenesis and protein synthesis.

## Results

### Increased expression of NAF1 is associated with poor prognosis in glioma patients

**Fig. 1:**
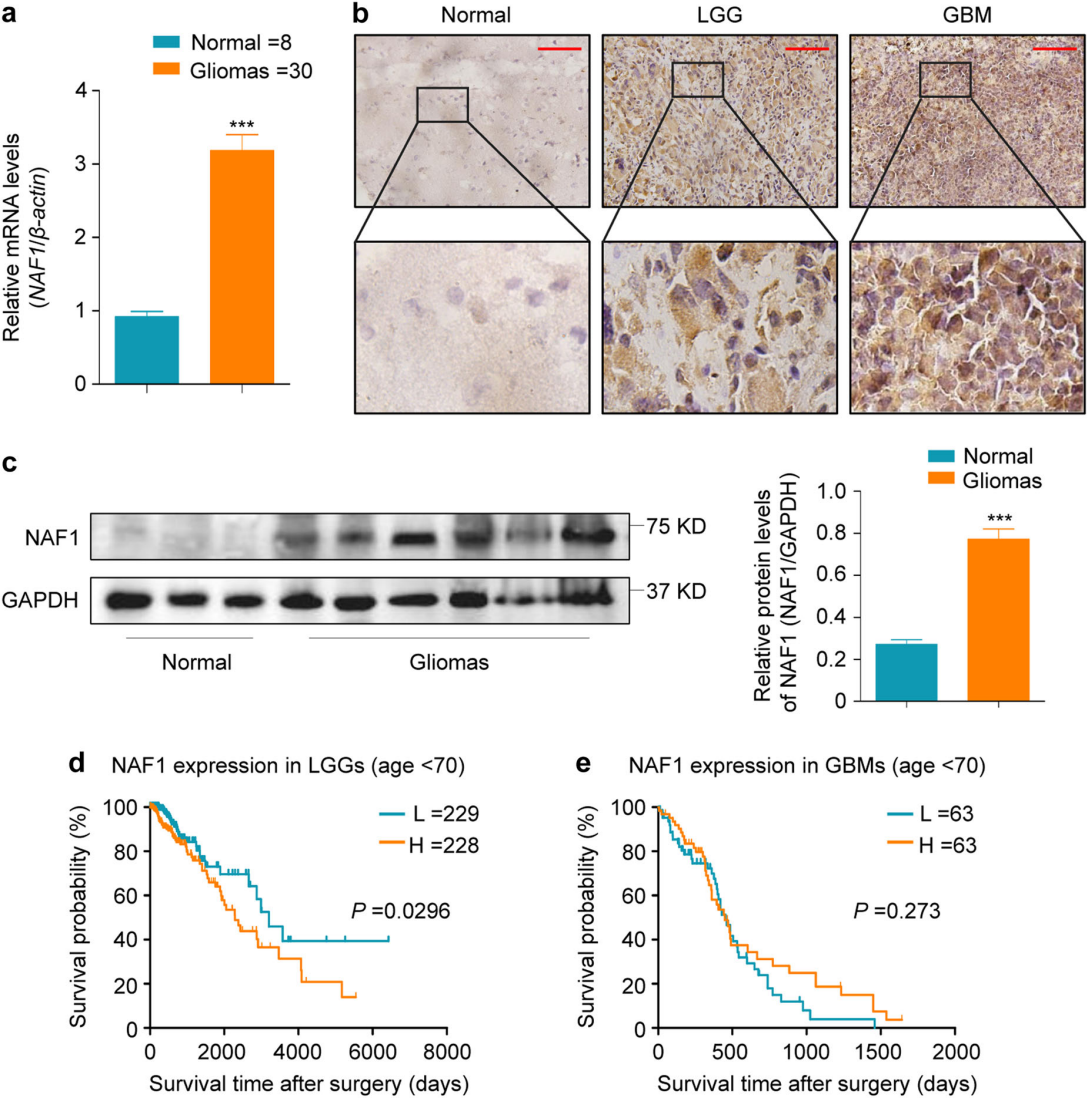
Association of increased expression of NAF1 with poor prognosis in glioma patients. **a** The qRT-PCR assay was carried out to assess mRNA expression of *NAF1* in normal brain tissues (N, *n* = 8) and gliomas (T, *n* = 30). *β-Actin* mRNA levels were used to normalize the *NAF1* expression. Data were presented as mean ± SD. ***P < 0.001 (*n* = 3). **b** The extent of specific staining (brown color) indicated the expression of NAF1. Upper images are representative images of immunohistochemistry (IHC) on histologic slides of normal controls, low-grade gliomas (LGGs), and glioblastoma (GBMs) using anti-NAF1 antibody. The lower images show magnifications of the area indicated by the black squares. Scale bars, 200 µm. **c** NAF1 expression was detected by western blot analysis in three cases of normal brain tissues and six cases of glioma patients with GAPDH as a loading control. Shown is representative of three independently preformed western blot experiments (left panels). Shown is the quantitative illustration of the levels of NAF1 proteins using densitometry (right panels). Data were shown as mean ± SD. ***P < 0.001 (*n* = 3). The relationship of increased expression of NAF1 with the survival of LGG patients (**d**) and GBM patients (**e**) in The Cancer Genome Atlas (TCGA) dataset

We first performed qRT-PCR, immunohistochemistry (IHC), and western blot assays to examine mRNA and protein levels of NAF1 in gliomas (*n* = 30) and normal brain tissues (*n* = 8; controls). The results indicated that, relative to control subjects, the mRNA and protein levels of NAF1 were clearly upregulated in gliomas (Fig. 1a–c). Next, we used The Cancer Genome Atlas (TCGA) dataset to evaluate the association of NAF1 expression with patient survival after excluding the patients over the age of 70 years, and found that there was a strong relationship between increased expression of NAF1 and poor overall survival in the patients with low-grade gliomas (LGGs) (*P* = 0.0296) (Fig. 1d), but not in those with glioblastomas (GBMs) (Fig. 1e).

Evidently, the overall survival of glioma patients can be affected by many important clinical and genetic factors, such as age, tumor grades, *ATRX* status, *IDH1/2* status, *telomerase reverse transcriptase* (*TERT*) status, and so on^16,17^. Therefore, we explored the correlation of *NAF1* expression with clinicopathologic and genetic characteristics in gliomas using the TCGA dataset. As shown in Supplementary Table S1, *NAF1* expression was significantly downregulated in the patients with LGGs (*P* = 0.005) and *ATRX* mutations (*P* = 0.001) compared to those with GBMs and wild-type ATRX, while was obviously associated with poor patient survival (*P* = 0.006). This was supported by the previous studies that glioma patients with LGGs and *ATRX* mutations generally had better prognosis than those with GBMs and wild-type ATRX^18^. In addition, we failed to find any relationship between *NAF1* expression and other clinicopathologic and genetic characteristics (Supplementary Table S1).

### *NAF1* is transcriptionally regulated by c-Myc, NRF2, and TERT in glioma cells

**Fig. 2:**
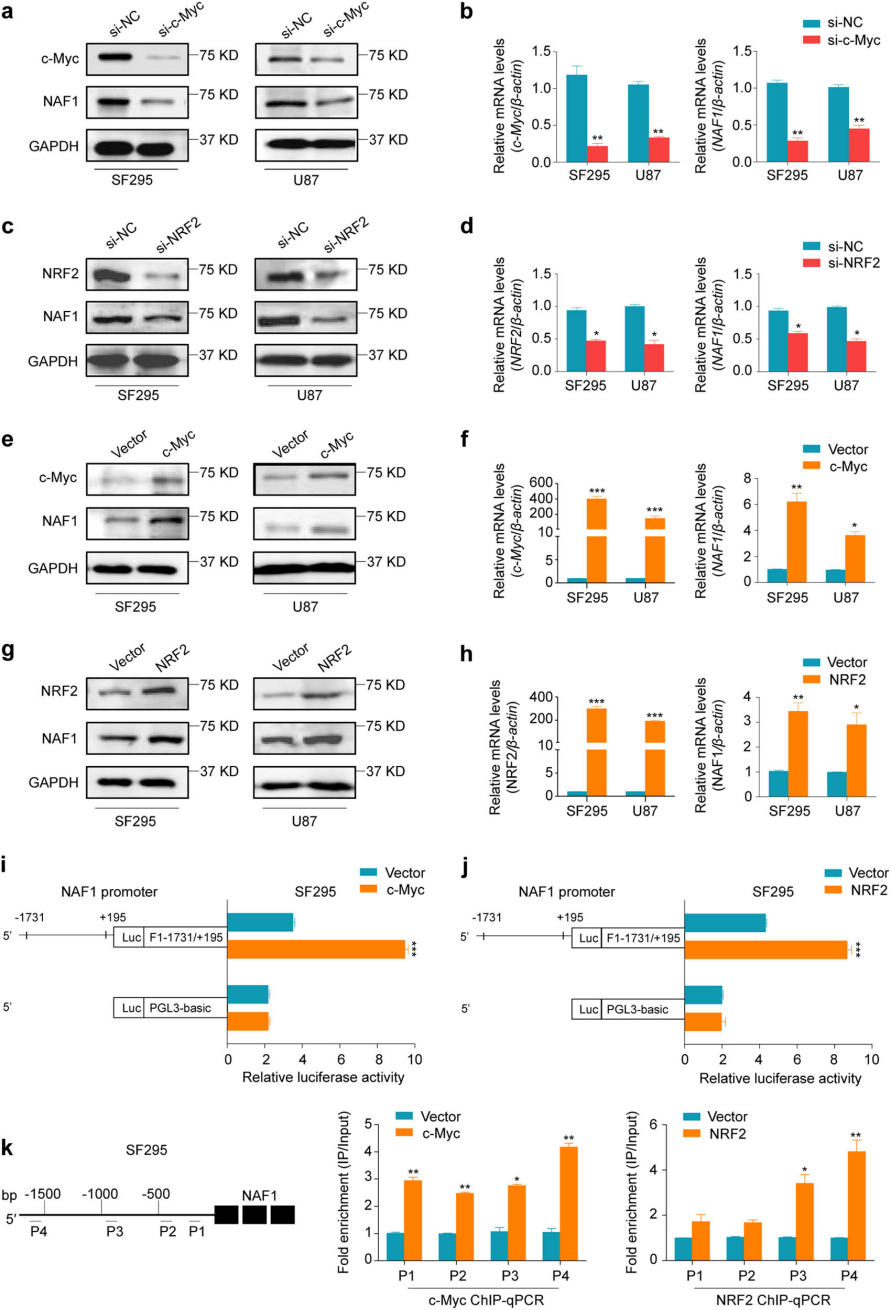
Transcriptional regulation of NAF1 by c-Myc and NRF2 in glioma cells. Upon knocking down of c-Myc (**a**, **b**) and NRF2 (**c**, **d**) in glioma cell lines SF295 and U87 using siRNAs, the protein and mRNA expression levels of NAF1, c-Myc, and NRF2 were measured by western blot and qRT-PCR assays. Shown is representative of three independently preformed western blot experiments. GAPDH and *β-actin* were used as the normalized controls for western blot and qRT-PCR assays, respectively. Data were shown as mean ± SD, *P < 0.05; **P < 0.01 (*n* = 3). Upon ectopic expression of c-Myc (**e**, **f**) and NRF2 (**g**, **h**) in glioma cell lines SF295 and U87, the protein and mRNA expression levels of NAF1, c-Myc, and NRF2 were determined by western blot and qRT-PCR assays. Shown is representative of three independently preformed western blot experiments. GAPDH and *β-actin* were used as the normalized controls for western blot and qRT-PCR assays, respectively. Data were shown as mean ± SD, *P < 0.05; **P < 0.01; ***P < 0.001 (*n* = 3). The Dual-Luciferase Reporter assay system was used to evaluate the impact of ectopic expression of c-Myc (**i**) and NRF2 (**j**) on the promoter activity of NAF1 in SF295 and U87 cells with the empty vector or control lentivirus as the controls. All the ratios of the Luc/Renilla activity were expressed as means ± SD. ***P < 0.001 (*n* = 3). **k** SF295 cells expressing c-Myc and NRF2 and control cells were subjected to ChIP-qPCR assays using corresponding primary antibodies. P1–P4 indicated four different regions of *NAF1* promoter (P1: −100/−26; P2: −519/−410; P3: −981/−543; P4: −1648/−1551) (left panel). Fold enrichment was expressed as mean ± SD (middle and right panels). *P < 0.05; **P < 0.01 (*n* = 3)

Given that induced promoter is one of the simplest and most effective ways to initiate gene expression, we attempted to predict transcription factors regulating promoter activity of *NAF1* using online tools (http://www.genecards.org and http://jaspar2014.genereg.net). Among numerous predicted transcription factors, we paid great attention to c-Myc and NRF2 because they have been widely studied in human cancers^19,20^, and demonstrated to play critical roles in malignant progression of gliomas^21,22^. Next, we explored the connection between their expression and *NAF1* expression in gliomas using TCGA dataset. As shown in Supplementary Fig. S1, the expression of *NAF1* was significantly positively connected with the expression of *c-Myc* and *NRF2*. In addition, the results showed that knocking down c-Myc and NRF2 in SF295 and U87 cells dramatically decreased mRNA and protein expression of NAF1 compared to the controls (Fig. 2a–d). On the other hand, overexpression of c-Myc and NRF2 in SF295 and U87 cells significantly upregulated NAF1 expressions relative to the controls (Fig. 2e–h). Altogether, our findings showed that *NAF1* is transcriptionally regulated by c-Myc and NRF2 in glioma cells.

To further explore whether c-Myc and NRF2 can regulate promoter activity of NAF1, we first cloned the promoter of NAF1 into a pGL3-Basic luciferase plasmid for the construction of the luciferase reporter plasmid pGL3-NAF1-Luc (−1731/+195). SF295 cells expressing c-Myc and NRF2 were then cotransfected with pGL3-NAF1-Luc or pGL3-Basic-Luc, and pRL-TK plasmids. The results indicated that ectopic expression of c-Myc and NRF2 were able to dramatically increase promoter activity of NAF1 compared to the controls (Fig. 2i, j). Next, we attempted to determine whether c-Myc and NRF2 directly bind to NAF1 promoter to regulate its promoter activity. Using specific c-Myc and NRF2 antibodies, we conducted the chromatin immunoprecipitation (ChIP) assay in SF295 cells expressing c-Myc and NRF2 and control cells, followed by the qPCR assay using primers specific to four different regions within *NAF1* promoter (Fig. 2k, left panel). As expected, compared to control cells, all four fragments within *NAF1* promoter (P1: −100/−26; P2: −519/−410; P3: −981/−543; P4: −1648/−1551) were strongly enriched in cells expressing c-Myc (Fig. 2k, middle panel), while two fragments (P3: −981/−543; P4: −1648/−1551) were clearly enriched in cells expressing NRF2 (Fig. 2k, right panel). Collectively, the data indicate that NAF1 is a direct target of c-Myc and NRF2.

In recent years, TERT promoter mutations have been frequently discovered in human cancers particularly in GBMs^23,24^. In addition, TERT has also been demonstrated to act as a transcriptional modulator, which regulates the activity of some major pathways such as the Wnt/β-catenin and NF-kB cascades^25^. Thus, we supposed that TERT may be involved in regulating *NAF1* transcription in glioma cells. To address this, we first determined whether NAF1 expression was regulated by TERT in glioma cells. The results showed that knocking down TERT in SF295 and U87 cells significantly downregulated NAF1 expression, while ectopic expression of TERT in these two cell lines dramatically upregulated NAF1 expression at both protein and mRNA levels (Supplementary Fig. S2a–d). Similarly, we demonstrated that TERT could bind to *NAF1* promoter and regulate its promoter activity by using the ChIP-qPCR assay and dual-luciferase reporter system (Supplementary Fig. S3 and S4). Altogether, our data show that NAF1 is also transcriptionally regulated by TERT.

### NAF1 promotes glioma cell growth and invasiveness in vitro

**Fig. 3:**
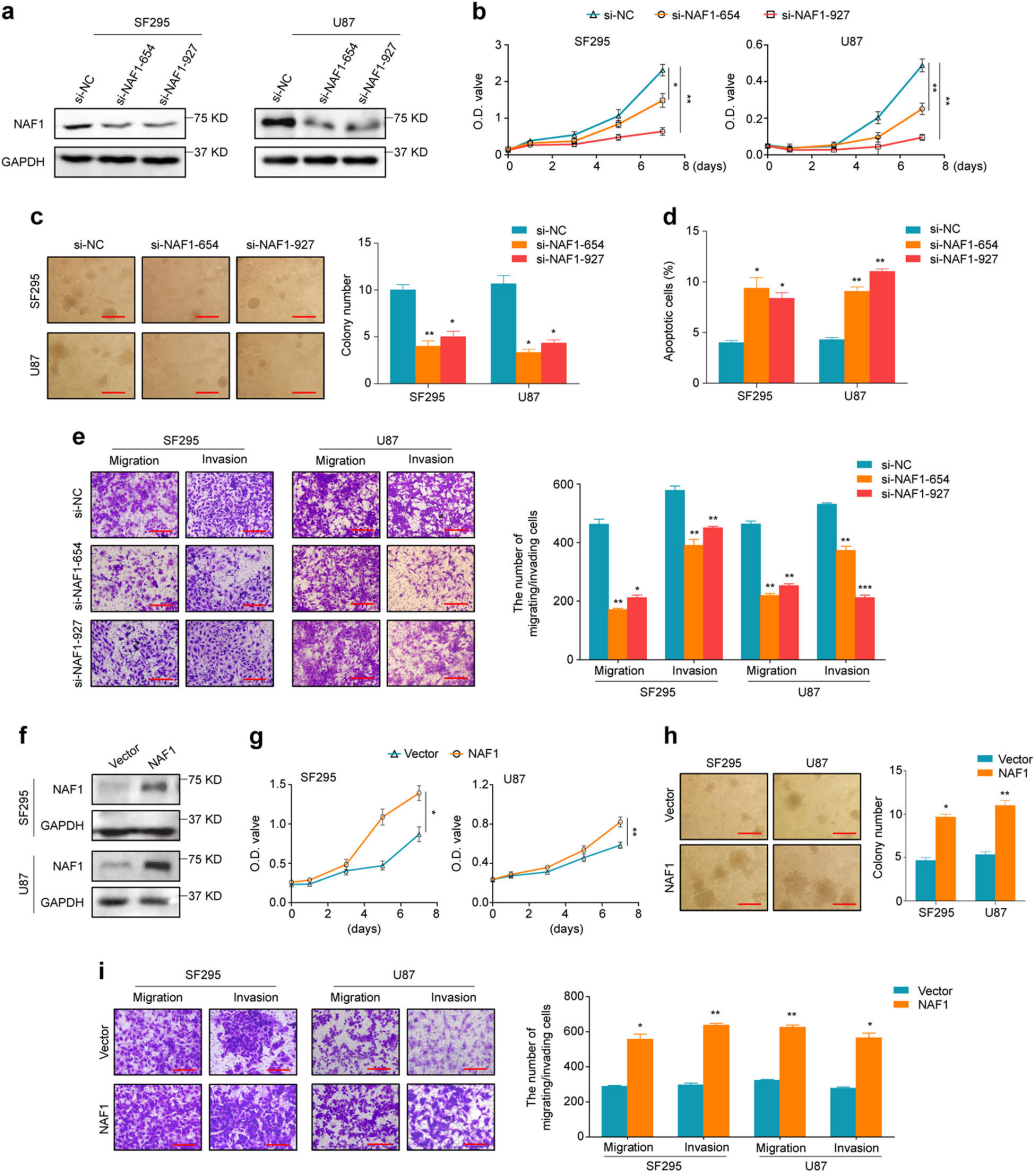
In vitro oncogenic activity of NAF1 in glioma cells. **a** Western blot analysis was performed to verify NAF1 depletion by two different siRNAs (si-NAF1-654 and si-NAF1-927) in SF295 and U87 cells with GAPDH as an endogenous control. **b** Cell viability upon NAF1 knockdown in SF295 and U87 cells was determined by the MTT assay. *P < 0.05; **P < 0.01 (*n* = 5). **c** Left panels show the representative images of colony formation in soft agar in SF295 and U87 cells upon NAF1 knockdown. Scale bars, 50 μm. Quantitative analysis of colony numbers (right panels). *P < 0.05; **P < 0.01 (*n* = 5). **d** Measurement of cell apoptosis in SF295 and U87 cells by flow cytometry. *P < 0.05; **P < 0.01 (*n* = 3). **e** The left panels are representative images of migrated/invaded cells upon NAF1 knockdown. The quantitative analysis of the number of migrated/invaded cells (right panels). Scale bars, 100 μm. *P < 0.05; **P < 0.01; ***P < 0.001 (*n* = 5). **f** Validation of ectopic expression of NAF1 in SF295 and U87 cell lines by western blot analysis with GAPDH as a loading control. **g** Cell viability upon NAF1 overexpression in SF295 and U87 cells was detected by the MTT assay, *P < 0.05; **P < 0.01 (*n* = 5). **h** The representative images of colony formation in soft agar upon NAF1 overexpression in SF295 and U87 cells (left panels). Scale bars, 50 μm. The right panels are the quantitative analysis of colony numbers. *P < 0.05; **P < 0.01 (*n* = 5). **i** The left panels are representative images of migrated/invaded cells upon NAF1 overexpression. Quantitative analysis of the number of migrated/invaded cells (right panels). Scale bars, 100 μm. Showing data as mean ± SD. *P < 0.05; **P < 0.01 (*n* = 5)

A series of loss- and gain-of-function experiments were performed in vitro to define the biological function of NAF1 in glioma. First, we validated inhibition efficiency of two different siRNAs targeting NAF1 (si-NAF1-654 and si-NAF1-927) in SF295 and U87 cells by qRT-PCR and western blot assays (Supplementary Fig. S5a and Fig. 3a). Next, we assessed the effect of NAF1 knockdown on glioma cell growth in vitro, and found that the proliferation of SF295 and U87 cells was significantly inhibited upon siRNA-mediated NAF1 knockdown compared to the controls (Fig. 3b). The soft agar colony-formation assay was conducted to further confirm the inhibitory effect on cell growth. As indicated in Fig. 3c, NAF1 knockdown obviously decreased the number and volume of colonies compared to the control. Then, we investigated the impact of NAF1 depletion on glioma cell apoptosis. The data showed that NAF1 depletion caused a dramatic increase in the percentage of apoptotic cells compared to the control, as shown in Fig. 3d. We also attempted to assess the impact of NAF1 depletion on the migration and invasion abilities of glioma cells. The results showed that NAF1 knockdown dramatically inhibited migration and invasion potential of glioma cells relative to control cells (Fig. 3e). On the contrary, as expected, ectopic expression of NAF1 in SF295 and U87 cells (Supplementary Fig. S5b and Fig. 3f) remarkably enhanced cell proliferation, colony formation, and migration and invasion, as shown in Fig. 3g–i. In addition, we observed the similar findings in other two glioma cell lines SHG44 and U251 (Supplementary Fig. S6). Altogether, these results indicated that NAF1 is a functional oncogene in glioma.

### NAF1 promotes ribosome assembly and protein synthesis in glioma cells

**Fig. 4:**
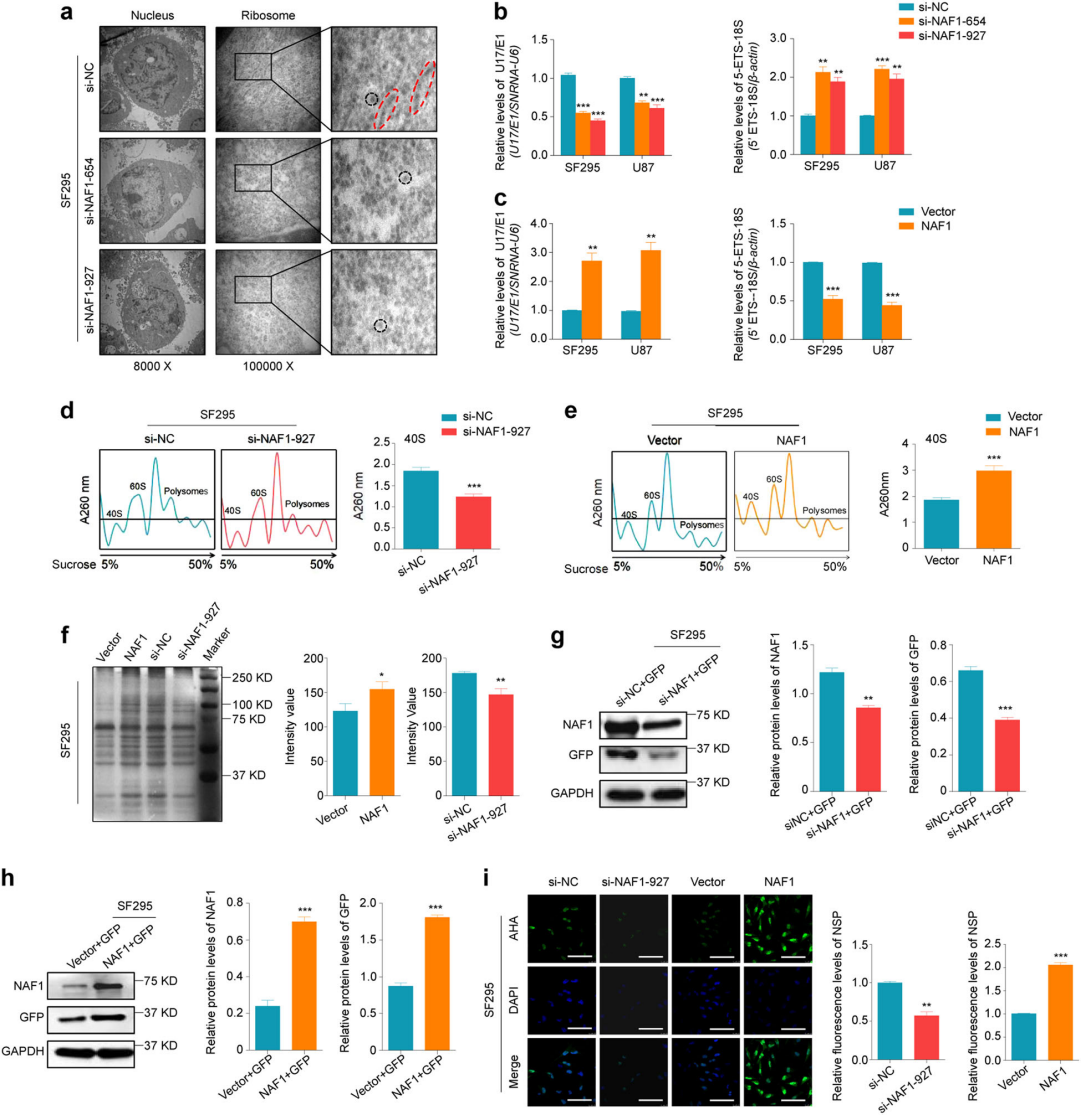
The effect of NAF1 on ribosome biosynthesis in glioma cells. **a** The electron tomography of the ribosomes. The nucleus is shown in the left panels (×8000), and the ribosomes are shown in the middle panels (×100,000). The magnifications of the area indicated by the black squares (right images). The red dotted circle indicates polyribosomes, while the black dotted circle indicates a single ribosome. **b**, **c** The expression of *U17 H/ACA* small nucleolar RNA (snoRNA) and 5′ETS-*18S rRNA* in SF295 and U87 cells were analyzed by the qRT-PCR assay. *U6 H/ACA* snoRNA and *β-actin* were used as endogenous controls. **P < 0.01; ***P < 0.001 (*n* = 3). **d**, **e** Sucrose density gradient analysis of 40S subunits in SF295 cells. The matched curves of the absorbance of different components of the ribosomes (left panels). The quantitative of abundance of 40S subunits (right panels). ***P < 0.001 (*n* = 3). **f** Total proteins extracted from the same amount of cells were visualized in SF295 cells by the PAGE-silver nitrate staining assay (left panel). The right panels are the quantification of the total density of proteins. *P < 0.05; **P < 0.01 (*n* = 3). **g**, **h** The synthesis ability of GFP proteins was evaluated in SF295 cells by western blot analysis. GAPDH was used as an endogenous control. Shown in the right panels are the quantitative illustration of expression levels of GFP proteins. **P < 0.01; ***P < 0.001 (*n* = 3). **i** Measurement of protein synthesis in SF295 cells. Images were obtained using a confocal microscope (left panels). Green color represents staining of newly synthesized proteins, and the nuclei were stained by Hoechst 33342 into blue. Scale bars, 200 μm. The right panels are the fluorescence quantification of Alexa Fluor 488 relative to DAPI in cells using ImageJ. Data are shown as mean ± SD. **P < 0.01; ***P < 0.001 (*n* = 5)

It is clear that efficient ribosome subunit biogenesis is pivotal to gene expression and plays an irreplaceable role in the life cycle of cells^5^. Moreover, alterations in ribosome biogenesis have been demonstrated to contribute to tumor initiation and progression^26,27^. Naf1p, the yeast homologue of mammalian NAF1, is necessary for ribosome biogenesis and yeast growth^28^. However, in humans, the physiological function of NAF1 and its association with the crucial biological processes during carcinogenesis remain uncertain; thus we speculate that NAF1 promotes glioma tumorigenesis and progression through regulating the assembly and function of ribosomes. First, we observed the ribosomes in SF295 cells by electron microscopy. As shown in Fig. 4a, si-NC-transfected cells possessed a cytoplasm overcrowded with ribosomes, and some of which formed longer and impact linear arrangements which were ready to translation, while si-NAF1-transfected cells showed an evenly distributed ribosomes and lesser polyribosome in cytoplasm. Although the integral measurement of the total ribosome number is an inappropriate readout as the amounts of ribosomes pre-existing and newly synthesized are very huge^29^, this phenomenon will inspire us to investigate the association of aberrant expression of NAF1 with ribosome synthesis.

Ribosomal biogenesis, the sophisticated and complex biological process, includes synthesis and processing of preribosomal RNAs, coordinated synthesis of ribosome protein, assembly and transport of ribosome^26^. As proved, Naf1p affects the accumulation of all box H/ACA snoRNAs and cell growth by interference of 18S pre-rRNA processing in the yeast^28^. Small nucleolar RNA SNORA73 (U17), the only one mammalian H/ACA RNP, mediates cleavage processing of the 5′ end of 18S rRNA into mature 18S rRNA^11,30^. It is noteworthy that a decrease of the quantities of hNaf1 (encoded by human *NAF1* gene) in the yeast is associated with a reduction in the steady levels of box H/ACA snoRNAs (including U17), scaRNAs, and telomerase RNA (TERC). In addition, NAF1 can deposit much more U17 than other H/ACA sno/scaRNAs in HeLa cell extracts by the co-immunoprecipitation (Co-IP) assay^31^. Thus, as mentioned above, we supposed that NAF1 may function on 18S pre-rRNA cleavage processing by affecting U17 expression, thereby modulating ribosomal biogenesis in glioma cells. To prove this, we designed the primers amplified out the products contain processed region and a part of invariant region to distinguish the precursor from the mature human 18S rRNA molecules. Next, the levels of U17 and 5′ETS-18S rRNA junction were analyzed by qRT-PCR in SF295 and U87 cells. As shown in Fig. 4b, relative to the control, NAF1 knockdown dramatically decreased U17 levels, while increased the levels of immature 18S rRNA in these two cell lines. Conversely, ectopic expression of NAF1 in SF295 and U87 cells dramatically increased U17 levels and decreased the levels of unprocessed 18S rRNA (Fig. 4c). Thus, we conclude that human NAF1 may facilitate the processing of 5′ETS-18S rRNA into mature 18S rRNA by enhancing the U17 accumulation.

In eukaryotes, the 18S rRNA is the only core rRNA in small 40S ribosomal subunit. Nonetheless, blocking of cleavage process delays or hinders rRNA maturation kinetics will lead to imbalanced ribosomal subunits (40S and 60S) in the cytoplasm^26^. To determine whether aberrant expression of NAF1 in glioma cells can perturb the accumulation of ribosomal 40S subunits, we performed the sucrose gradient centrifugation. We observed an obvious decrease of 40S ribosomal subunits accumulation in NAF1-depletion cells (Fig. 4d), while the opposite results were found in the NAF1-transfected cells compared with the controls (Fig. 4e). It is conceivable that NAF1 may be required for these processing events. This will result in the elimination of the 5′ETS spacer of the pre-rRNA, then producing the 18S pre-rRNA and 40S ribosomal subunits.

After the formation, the 40S and 60S ribosomal subunits are transported from the nucleolus to the cytoplasm, via the nucleoplasm, for ribosome assembly of 80S ribosomal subunits and protein synthesis. It is well known that tumor cells are characterized by rapid cell proliferation and abundant protein synthesis^26^. Thus, we speculate that NAF1 should be involved in modulating protein synthesis ability of ribosomes. As shown in Fig. 4f, relative to the control, we discovered that overexpression of NAF1 obviously increased global protein concentration, while NAF1 knockdown decreased global protein levels when total proteins of the same numbers of SF295 cells were extracted and run the PAGE-silver staining assay. Next, we attempted to determine the effect of NAF1 on protein synthesis ability through observing the production of exogenous GFP proteins in glioma cells. The result showed that the NAF1 knockdown in SF295 cells evidently inhibited protein synthesis of exogenous GFP, while ectopic expression of NAF1 increased the levels of exogenous GFP compared to the controls (Fig. 4g, h). We further proved the above conclusions by employing metabolic labeling of proteins with the noncanonical amino acid, azidohomoalanine (AHA), which is analogs of methionine (Met) to quantify newly synthesized proteins (NSPs)^32^. As shown in Fig. 4i, NAF1 knockdown in SF295 cells exhibited weaker fluorescence intensity, indicating a decreased ability of protein synthesis, while NAF1 re-expression exhibited brighter fluorescence intensity, indicating an enhanced ability of protein synthesis. In conclusion, our data showed that NAF1 promotes glioma tumorigenesis and progression probably through enhancing ribosome assembly and protein synthesis.

**Fig. 5:**
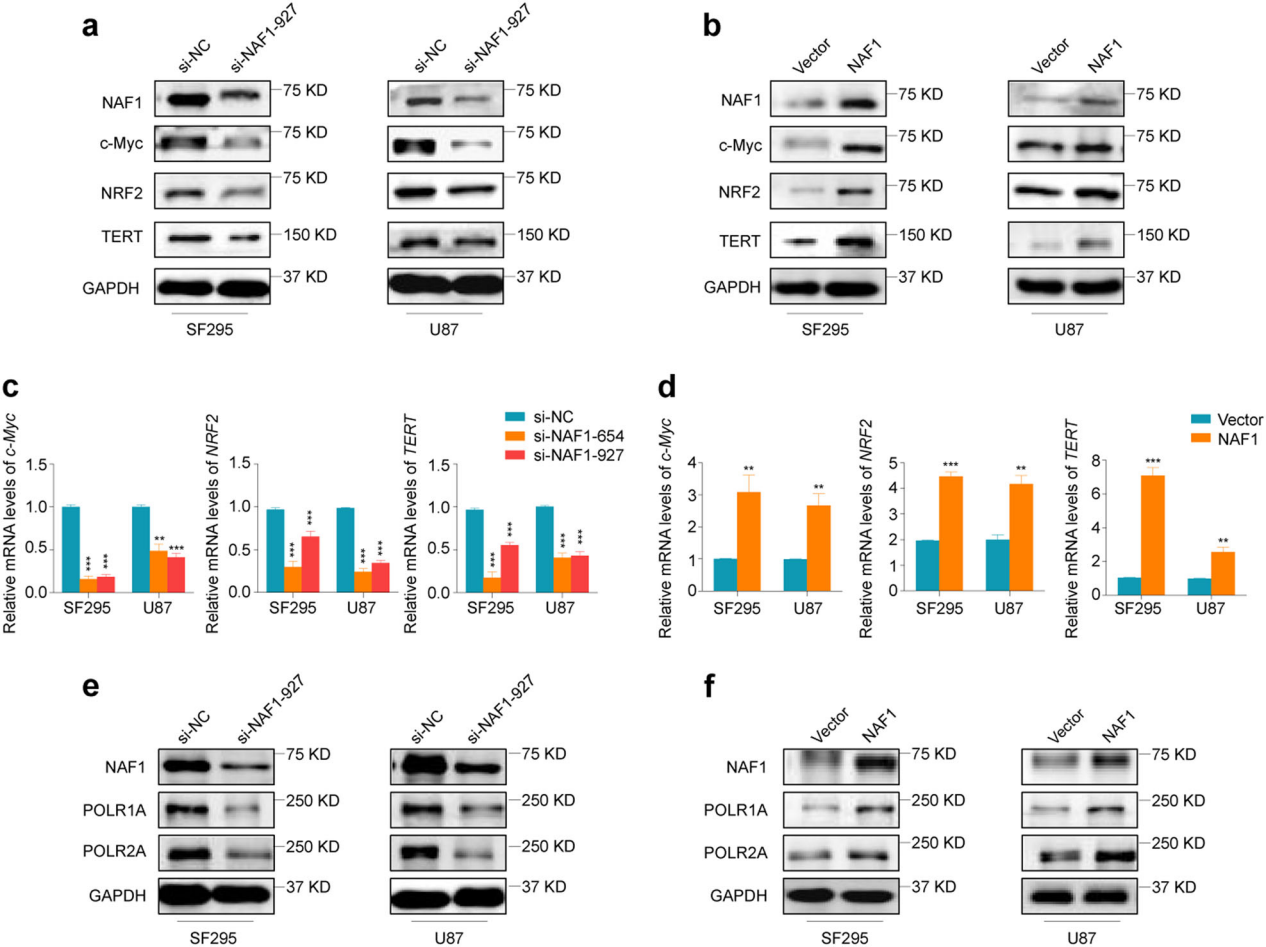
NAF1 promotes the expression of c-Myc, NRF2, TERT, POLR1A, and POLR2A in glioma cells. **a**, **b** Protein expression levels of c-Myc, NRF2, and TERT upon NAF1 knockdown or ectopic expression in SF295 and U87 cells were determined by western blot analysis with GAPDH as a loading control. **c**, **d** The qRT-PCR assay was carried out to detect mRNA expression of *c-Myc*, *NRF2*, and *TERT* upon depletion or overexpression of NAF1 in SF295 and U87 cells. *β-Actin* was used as an endogenous control. Data were shown as mean ± SD. **P < 0.01; ***P < 0.001 (*n* = 3). **e**, **f** Western blot analysis was used to evaluate protein expression of POLR1A and POLR2A upon knockdown or ectopic expression of NAF1 in SF295 and U87 cells. GAPDH was used as an endogenous control. The western blot is representative of three independently preformed experiments

Alterations in ribosome production lead to the readjustment in total translation, or changes in translation of specific mRNAs, which are linked to the control of cell growth and survival^26^. Thus, we suppose that aberrant expression of NAF1 will impact the translation of key molecules in malignant progression of gliomas such as c-Myc, NRF2, and TERT, which have been demonstrated to regulated *NAF1* transcription as mentioned above. Indeed, we found that NAF1 depletion in SF295 and U87 cells dramatically decreased protein levels of c-Myc, NRF2, and TERT (Fig. 5a), while ectopic expression of NAF1 expectedly increased their protein levels relative to the controls (Fig. 5b). However, we surprisingly found that knockdown or ectopic expression of NAF1 in glioma cells also impacted mRNA levels of *c-Myc*, *NRF2*, and *TERT* (Fig. 5c, d), suggesting that there may exist unknown mechanisms of NAF1 modulating gene transcription. It is clear that RNA polymerase II (RNA Pol II) is the core transcriptase capable of synthesizing the precursors of mRNAs, most snRNAs and microRNAs^33^. Moreover, the synthesis of 45S rRNA, a major RNA section, is the first event in ribosome biogenesis, and this process is dependent on RNA polymerase I (RNA Pol I)^34^. Thus, we suppose that NAF1 is associated with the regulation of gene transcription probably by affecting protein synthesis of RNA Pol I and II. As shown in Fig. 5e, NAF1 knockdown dramatically decreased protein levels of the core subunit of RNA pol I, POLR1A, and the key subunit of RNA pol II, POLR2A, in SF295 and U87 cells. Conversely, ectopic expression of NAF1 in these two cell lines increased their protein levels (Fig. 5f). In addition, a previous study has indicated that there is a direct interaction between yeast Naf1p and the C-terminal domain of POLR2A^28^. Thus, we next attempted to determine whether there may be similar interaction between them in mammalian cells. However, we failed to find the combination between POLR2A and NAF1 in SF295 cells (Supplementary Fig. S7). Collectively, these observations suggest that NAF1 may regulate gene transcription such as 45S rDNA, *c-Myc*, *NRF2,* and *TERT* through impacting the biogenesis of RNA Pol I and II.

### NAF1 lengthens telomere length in glioma cells

Aberrant telomere length has been considered as one of the hallmarks for cell proliferation and human cancers, including glioma^35,36^. TERC and TERT are the core components of the vertebrate telomerase H/ACA RNP, which is the key factor for telomere maintenance^37–39^. Recently, there have been studies showing that SNPs located in *NAF1* gene may be linked to the modulation of telomere length through impacting its expression^14^. Moreover, we have demonstrated the impact of NAF1 on the mRNA and protein expression of TERT as mentioned above. Therefore, we hypothesize that NAF1 may participate in telomere length maintenance in glioma cells. These data indicated that NAF1 reduction significantly decreased the levels of TERC and shortened telomere length in SF295 and U87 cells (Supplementary Fig. S8a, b), while ectopic expression of NAF1 elevated the levels of TERC and lengthened telomere length (Supplementary Fig. S8c, d). Altogether, our data suggest that NAF1 lengthens telomere length through regulating TERT at both transcription and translation levels and TERC at transcription levels in glioma cells.

### NAF1 depletion triggers ribosome stress and relocalization of ribosomal proteins

**Fig. 6:**
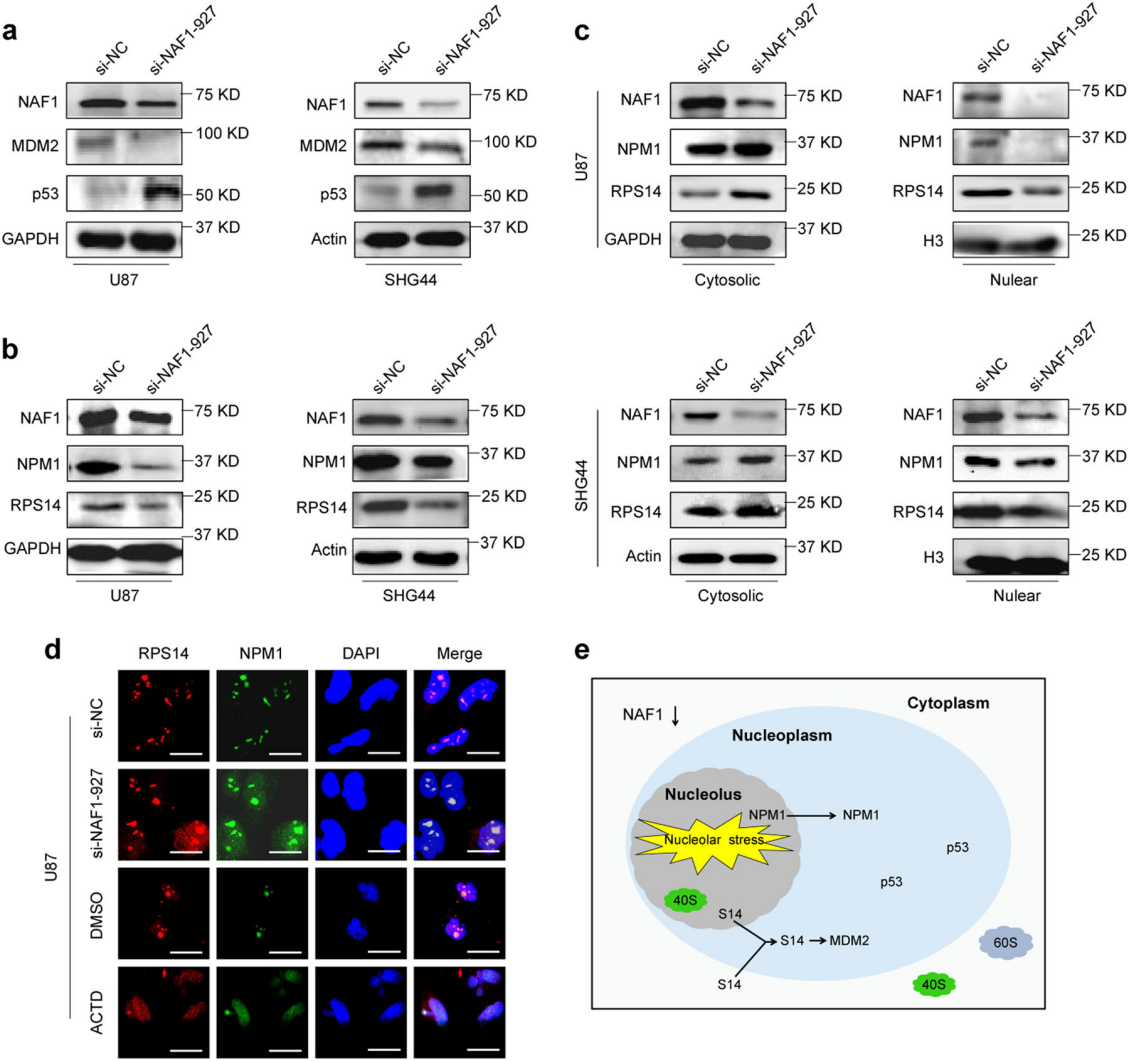
The ribosome stress is triggered by NAF1 depletion in glioma cells. **a** Western blot analysis of MDM2 and p53 proteins upon NAF1 knockdown in U87 and SHG44 cells. Using GAPDH and Actin as loading controls, and the western blot is representative of three independently preformed experiments. **b**, **c** Western blot analysis was conducted to evaluate the effect of the NAF1 depletion on the expression and cellular localization of the NPM1 and RPS14 in U87 and SHG44 cells. Using GAPDH, Actin and histone H3 as loading controls, and the western blot is representative of three independently preformed experiments. **d** The effect of NAF1 knockdown on subcellular localization of RPS14 and NPM1 in U87 cells was investigated by the immunofluorescence assay. Red color represents nuclear and cytoplasmic staining of RPS14; green color represents nuclear and cytoplasmic staining of NPM1; blue color represents Hoeschst 33342 staining for nuclei; fluorophores in the three-color overlay are labeled to the rightmost. Actinomycin D treatment was used as positive control, while DMSO treatment was used as the vehicle control. Scale bars, 500 μm. The experiments were performed in triplicate. **e** Working model of NAF1 depletion triggering ribosome stress. Briefly, upon NAF1 reduction, NPM1 is dissociated from nucleolar to nucleoplasm. As well, the waved circle indicates the RPS14 which is released from breaking down of cytoplasmic or nucleolar ribosomes, entering the nucleoplasm to interact with MDM2. This will result in p53 reactivation by blocking MDM2-p53 feedback loop

In eukaryotes, the biogenesis of cytoplasmic ribosomes is a complex process that takes place mainly in the nucleolus^40^. However, the perturbation of any step in ribosomal biogenesis, such as the disturbance of rRNA synthesis and processing, ribosomal protein synthesis, and ribosome assembly can induce nucleolar/ribosomal stress^41^. Under such stress, ribosomal biogenesis is inhibited, and NPM1 (the most typical hallmark of nucleolar stress) is released from nucleolar to nucleoplasm^42^. As well, free forms of ribosomal proteins (RPLs and RPSs) are released into the nucleoplasm to reactivate p53 by blocking the classical MDM2-p53 feedback loop, which guarantees the process of ribosomal biogenesis^43–45^. Our data have demonstrated that NAF1 can accelerate 18S rRNA processing and ribosome biogenesis, even protein synthesis. Thus, we speculate that NAF1 depletion in glioma cells may result in nucleolar/ribosomal stress. Given that SF295 and U251 cells harbor mutant p53 (https://cancer.sanger.ac.uk/cosmic), thus we chose U87 and SHG44 cells carrying wild-type p53 for the following studies. Expectedly, NAF1 knockdown in U87 and SHG44 cells clearly decreased MDM2 expression, and subsequently elevated p53 expression (Fig. 6a). Meanwhile, NAF1 depletion also decreased the expression of ribosome small subunit RPS14 and nucleophosmin NPM1 in these two cell lines (Fig. 6b). Considering that treatment of the cells with low dose of Actinomycin D (Act D) can trigger ribosomal stress through impairment of ribosomal biogenesis^41^, thus we treated glioma cells with 5 nM Act D as a positive control of ribosomal stress. By western blot and immunofluorescence assays, we found that NAF1 knockdown caused RPS14 and NPM1 translocating from nucleolus to nucleoplasm (Fig. 6c, d). Taken together, these findings indicate that NAF1 reduction may trigger ribosome stress, and subsequently causes translocation of RPS14 and NPM1 into nucleoplasm, thereby reactivating p53 signaling by blocking MDM2 (Fig. 6e).

### NAF1 promotes tumor growth in nude mice

**Fig. 7:**
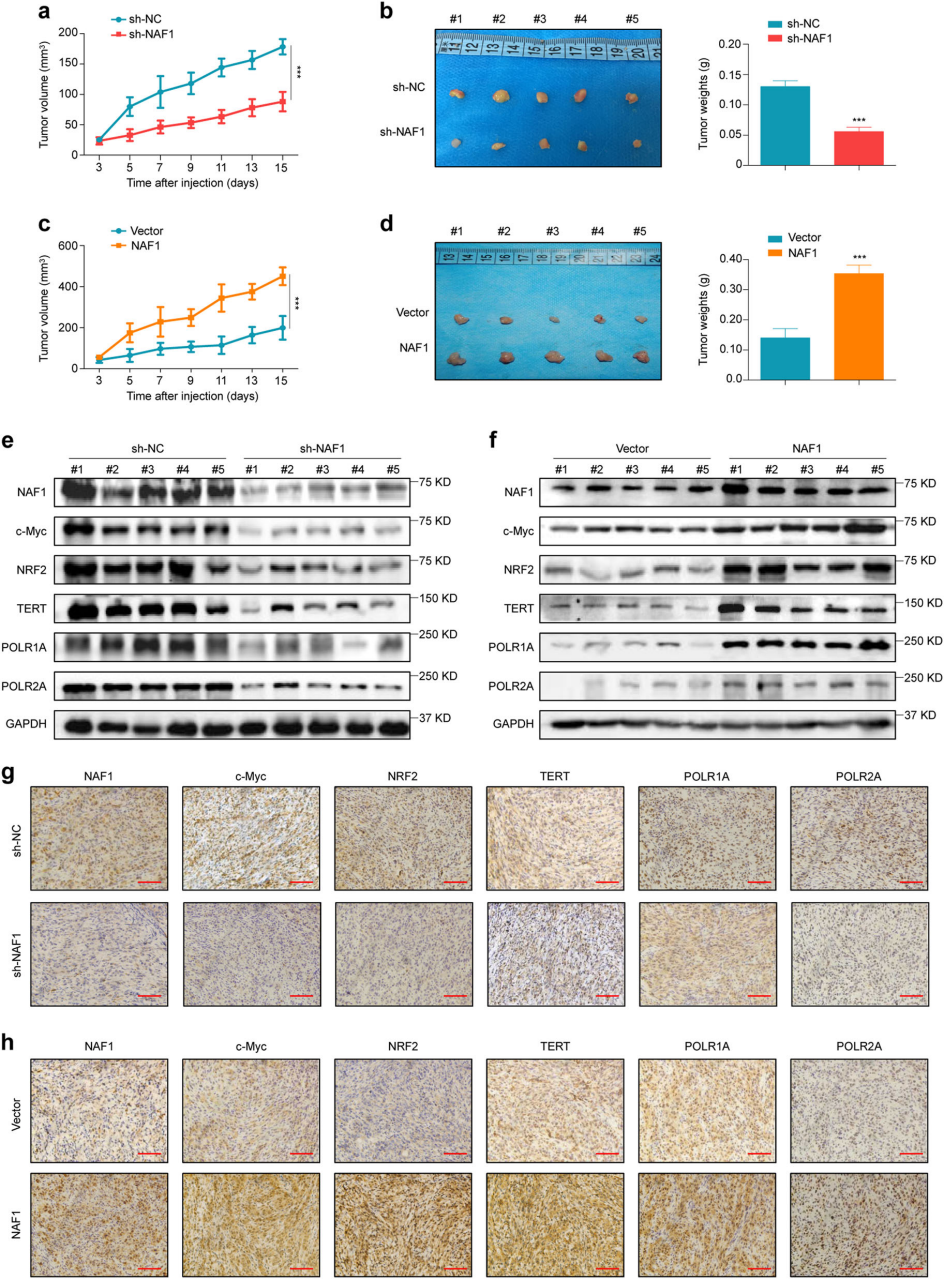
Stimulation of xenograft tumor growth by NAF1. **a** Comparison of tumor growth curves between U87 cells stably knocking down NAF1 and control cells in nude mice. Data were expressed as mean ± SD. ***P < 0.001 (*n* = 5/group). Day 0 indicates time point of tumor cell injection. **b** Images of dissected tumors from nude mice are presented in left panels. Histogram represents the average weight of xenograft tumors from control and NAF1-knockdown groups (right panels). ***P < 0.001 (*n* = 5*/*group). **c** Comparison of tumor growth curves between U87 cells stably expressing NAF1 and control cells in nude mice. Data were expressed as mean ± SD. ***P < 0.001 (*n* = 5*/*group). Day 0 represents time point of tumor cell injection. **d** Images of dissected tumors from nude mice are presented in left panels. Histogram represents the average weight of xenograft tumors from NAF1-overexpressing and control groups (right panels). ***P < 0.001 (*n* = 5*/*group) **e**, **f** Xenograft tumors were homogenated, lysed, and then subjected to western blot analysis using the indicated antibodies with GAPDH as a loading control. **g**, **h** Representative xenograft tumor sections from NAF1-knockdown, NAF1-overexpressing, and corresponding control groups were subjected to IHC staining. Scale bars, 200 μm

We also tested tumorigenic potential of NAF1 in nude mice. The results showed that xenograft tumors induced by U87 cells stably knocking down NAF1 grew slowly and exhibited a dramatic reduction of tumor volume and weight relative to control tumors (Fig. 7a, b). Conversely, the tumors induced by U87 cells stably expressing NAF1 grew more rapidly and showed larger mean tumor volume and weight than control tumors (Fig. 7c, d). Meanwhile, the IHC assay was conducted to detect the protein levels of Ki-67 (a well-known proliferative marker) to evaluate the proliferative ability of the above xenograft tumors. As shown in Supplementary Fig. S9, we found a lower percentage of Ki-67-positive cells in the tumors stably knocking down NAF1, while an increased number of Ki-67 cells in the tumors stably expressing NAF1 compared to control tumors.

Next, we further verified the regulatory effect of NAF1 on the expression of the above key molecules such as c-Myc, NRF2, TERT, POLR1A, and POLR2A in xenograft tumors. Using western blot analysis, we found that the protein level of NAF1 was dramatically decreased in the tumors stably knocking down NAF1 relative to control tumors (Fig. 7e and Supplementary Fig. S10a). Correspondingly, the expressions of c-Myc, NRF2, TERT, POLR1A, and POLR2A were also decreased in the NAF1-knockdown tumors compared to control tumors. However, NAF1 expression was significantly increased in the tumors stably expressing NAF1 compared with control tumors, and the expressions of c-Myc, NRF2, TERT, POLR1A, and POLR2A were correspondingly upregulated in the NAF1-overexpressing tumors compared to control tumors (Fig. 7f and Supplementary Fig. S10b). These results were further supported by the IHC assays (Fig. 7g, h and Supplementary Fig. S10c, d).

**Fig. 8:**
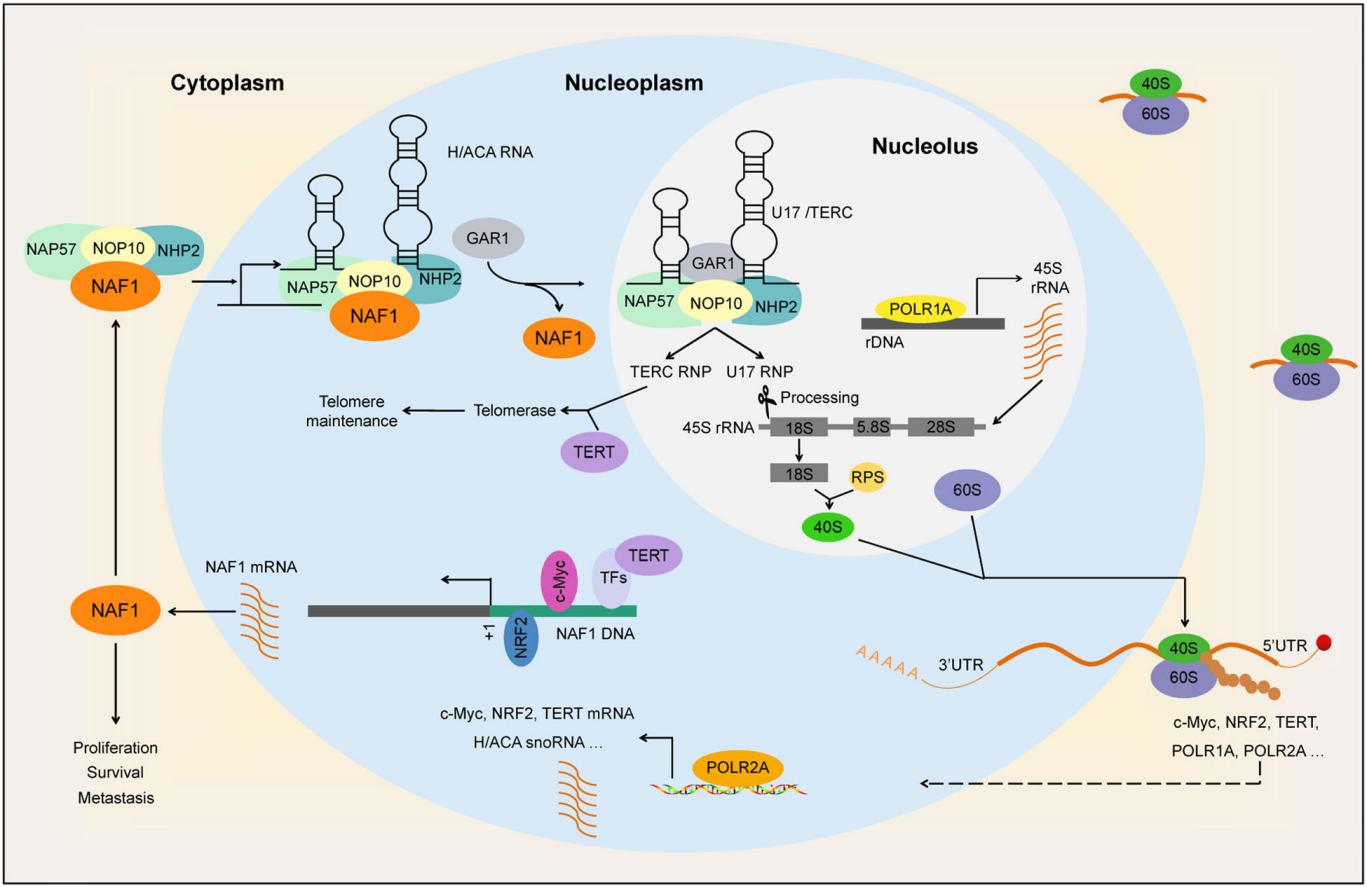
A schematic model for NAF1 promoting glioma tumorigenesis and progression through enhancing ribosome biosynthesis. NAF1 plays a crucial role in maintaining the yield of mature H/ACA RNPs. During malignant transformation of glioma cells, increased expression of NAF1 promotes U17 snoRNA processing, 18S rRNA maturation, and the assembly of 40 S subunits, thereby enhancing protein synthesis, including some key molecules associated with malignant progression of gliomas, such as c-Myc, NRF2, TERT, POLR1A, and POLR2A. Meanwhile, c-Myc, NRF2, and TERT in turn transcriptionally upregulate *NAF1* expression, while POLR1A and POLR2A also can active the transcription of *45S rRNA*, *c-Myc*, *NRF2*, *TERT*, and *H/ACA* snoRNA. These observations indicate that there exist positive feedback loops between NAF1 and these key molecules. In addition, NAF1 can maintain telomere length by increasing the levels of TERT and TERC at transcriptional or post-transcriptional levels. Altogether, these molecular events will contribute to glioma tumorigenesis and progression

Given the above, we propose a model to illustrate molecular mechanism of NAF1 promoting glioma tumorigenesis and progression (Fig. 8). During ribosome biogenesis, high expression of NAF1 enhances the assembly of 40S subunits and protein synthesis ability through increasing the levels of U17 snoRNA and accelerating 18S rRNA maturation in gliomas cells. This will enhance the translation of a number of key genes associated with malignant progression of gliomas, such as c-Myc, NRF2, TERT, POLR1A, and POLR2A. In turn, c-Myc and NRF2 can transcriptionally regulate *NAF1* expression through binding to its promoter, while TERT acts as a transcriptional coactivator to enhance *NAF1* transcription through indirectly binding to its promoter. Meanwhile, POLR1A and POLR2A may also promote ribosome biogenesis and NAF1 expression through transcriptionally regulating the expression of 45S rRNA, c-Myc, NRF2, TERT, and H/ACA snoRNA. Thus, there may be positive feedback loops between NAF1 and these key molecules in glioma cells. In addition, NAF1 may also maintain telomere length via modulating the expression of TERT and TERC. Collectively, NAF1 is crucial for ribosome biogenesis, and contributes to malignant phenotypes of glioma cells such as proliferation, survival, and metastasis.

## Discussion

NAF1 is required for the assembly of H/ACA box small nucleolar RNP^12^, and there is evidence showing the importance of Naf1p in cell growth in yeast^28^. However, its role in human cancers including glioma still has not been elucidated until now. In this study, we first proved that NAF1 was significantly elevated in gliomas relative to normal brain tissues, and found the correlation of increased expression of NAF1 with poor prognosis in LGG patients. Second, we discovered that NAF1 depletion in glioma cells significantly inhibited cell proliferation, colony formation, migration, invasion, and tumorigenic ability in nude mice, and induced cell apoptosis. Conversely, ectopic expression of NAF1 obviously enhanced malignant phenotypes of glioma cells, indicating that NAF1 is a functional oncogene in gliomas.

To clarify the mechanism underlying NAF1 upregulation in gliomas, we predicted and identified two important transcription factors c-Myc and NRF2 probably regulating *NAF1* transcription. Our data showed that NAF1 expressions were decreased upon c-Myc or NRF2 knockdown in glioma cells, while the mRNA and protein levels of NAF1 were increased upon overexpression of c-Myc or NRF2, suggesting that NAF1 may be a potential target of c-Myc and NRF2. This was further confirmed by the results from the dual-luciferase reporter and ChIP assays. Besides, there are studies showing that TERT acts as a transcriptional coactivator^25^. Thus, we speculate that TERT may also be involved in regulating *NAF1* transcription, as indicated by our data that TERT knockdown downregulated NAF1 expression, while ectopic expression of TERT upregulated its expression. Collectively, these results indicate that c-Myc, NRF2, and TERT can transcriptionally regulate NAF1 in glioma cells.

The infinite proliferation of cancer cells is closely associated with the changes in ribosome production and function^27^. It is well known that NAF1 is required for H/ACA snoRNP maturation, thereby regulating ribosome biosynthesis in eukaryotes^10,12,46^. Thus, we speculate that NAF1 promotes glioma tumorigenesis and progression probably through enhancing ribosome biosynthesis. Expectedly, our data presented that NAF1 ectopic expression in glioma cells dramatically accelerated 18S rRNA processing and 40S ribosome synthesis through increasing the levels of the scissor U17 H/ACA snoRNA, while NAF1 depletion in glioma cells obviously impaired the removal of 5′ETS of pre-rRNA, the maturation of 18S rRNA and the formation of 40S ribosomal subunit. Most importantly, by visualizing the distribution of AHA-labeled newly synthesized proteins, we demonstrated that NAF1 indeed enhanced protein synthesis through promoting the ability of ribosome biosynthesis in glioma cells. This was also supported by our data that NAF1 positively regulated protein expression of c-Myc, NRF2, TERT, POLR1A, and POLR2A. These data indicate that there may exist positive feedback loops between NAF1 and numerous key molecules associated with glioma initiation and progression.

Given the involvement of NAF1 in ribosome biosynthesis, and that the perturbation of any step in ribosomal biogenesis can trigger ribosomal stress^41^, we speculate that NAF1 reduction will cause this molecular event. As expected, our data showed that NAF1 knockdown in wild-type p53 glioma cells promoted the translocation of RPS14 and NPM1 into nucleoplasm, and subsequently reactivated p53 signaling by inhibiting MDM2, which is one of major characteristics of ribosome stress^43^. However, the exact mechanism of NAF1 depletion triggering ribosome stress remains largely unclear. Given the above, our findings indicate that NAF1 depletion in wild-type p53 glioma cells not only can attenuate ribosomal biosynthesis but also reactivate p53 signaling by blocking MDM2-p53 loop, thereby inhibiting malignant phenotypes of glioma cells.

It is clear that cancer cells have to maintain telomere length to counteract telomere shortening during tumorigenesis^35^. In recent years, inactivation or polymorphisms of *NAF1* gene have been reported to be implicated in telomere length maintenance in human diseases including cancer^15^. To be consistent with this, our data demonstrated that knocking down NAF1 in glioma cells obviously shortened telomere length, while ectopic expression of NAF1 significantly lengthened telomere length. Mechanistically, NAF1 maintains telomere length through regulating two major components of telomerase, TERT and TERC^37^, at transcription and/or translation levels, thereby contributing to malignant progression of gliomas.

In summary, we find that NAF1 is highly expressed in gliomas, and the increased expression of NAF1 is closely connected with poor patient survival. Through a series of systematic in vitro and in vivo studies, we demonstrate that NAF1 is a functional oncogene, and there may form positive feedback loops between NAF1 and numerous key molecules associated with malignant progression of gliomas via the regulation of ribosome biosynthesis. In addition, we find that NAF1 depletion can trigger ribosome stress, not only impairing ribosomal biosynthesis but also reactivating p53 signaling via blocking MDM2. Thus, NAF1 may be used as a valuable prognostic marker and even a potential therapeutic target for gliomas.

## Materials and methods

### Clinical samples and human glioma cell lines

Normal brain tissues (*n* = 8; form the patients with cerebral contusion and laceration) and gliomas (*n* = 30) were randomly collected from the First Affiliated Hospital of Xi’an Jiaotong University between January 2004 and September 2013. These patients did not receive preoperative chemotherapy or radiotherapy, and had signed an informed consent. More than two senior neuropathologists confirmed the histopathological diagnosis according to the classification of World Health Organization (WHO). This research was approved by ethics committee of Xi’an Jiaotong University.

Human glioma cell lines SF295 and SHG44 were purchased from the Cell Bank of Animal Laboratory Center of Zhongshan University (Guangzhou, China). U87 and U251 were obtained from the ATCC. These cell lines were routinely cultured at 37 °C in RPMI 1640 or DMEM (Gibco, Grand Island, NY) with 10% fetal bovine serum (FBS; Hyclone, Logan, UT). In addition, the Cell ID System (Promega) was used to perform the short tandem repeat (STR) DNA profiling of cell lines. Meanwhile, we used the one-step Quickcolor Mycoplasma Detection Kit (Shanghai Yise Medical Technology Co., Ltd.) to demonstrate that these cell lines were not contaminated by mycoplasma. In some experiments, cells were treated with Actinomycin D (HY-17559, MedChem Express, NJ) at the indicated concentrations and times, and using the same volume of the vehicle as the control.

### RNA extraction and quantitative RT-PCR (qRT-PCR)

Trizol reagent (Takara Inc., Dalian, China) and the PrimeScript RT reagent Kit (Takara Inc., Dalian, China) were used to isolate total RNA from the tissues or cell lines and make cDNA following the manufacturer’s instructions, respectively. We carried out qRT-PCR assays on a CFX96 Thermal Cycler DiceTM real-time PCR system (Bio-Rad Laboratories, Inc., CA) using SYBR Premix Ex TaqTM (Takara Inc., Dalian, China). Each sample was run in triplicate, and relative mRNA levels of each gene were normalized to β-actin or SNRNA-U6 cDNA. Supplementary Table S2 summarizes the primer sequences.

### Immunohistochemistry (IHC)

Using the IHC assay, we evaluated the expression levels of NAF1, c-Myc, NRF2, TERT, POLR1A, POLR2A, and Ki67 proteins in the xenograft tumors. The detailed protocol was performed as described previously^47^.

### Western blot analysis

Cells were lysed in prechilled RIPA buffer containing protease inhibitors, and equal amounts of protein lysates were separated by 10% SDS-PAGE and transferred to PVDF membranes (Roche Diagnostics, Mannheim, Germany). Next, we incubated the membranes with the indicated primary antibodies (Supplementary Table S3) at 4 °C overnight. After incubation the membranes with species-specific HRP-conjugated secondary antibodies (ZSGB-BIO, Beijing, China), we used the Western Bright ECL detection system (Advansta, CA) to visualize the immunoblotting signals.

### Expression plasmids, short interfering RNAs (siRNAs) and lentivirus transfection

The empty vector pcDNA3.1(-)A was purchased from Yingrun biotechnology, Co., Ltd. (Changsha, China). To construct NAF1 expression plasmid, the corresponding open reading frames (ORFs) with stop codon were amplified and then inserted into the mammalian expression vector pcDNA3.1(-) (Invitrogen, Grand Island, NY), named as pcDNA3.1-NAF1. Supplementary Table S4 summarizes the primers used for plasmid construction. NC16 pCDNA3.1 FLAG NRF2 (Plasmid #36971), pCDNA-3xHA-hTERT (Plasmid #51637), and corresponding empty vector were obtained from Addgene (Cambridge, MA). We transfected cells with the indicated constructs using X-tremeGENE HP DNA Transfection Reagent (Invitrogen, Grand Island, NY) at 70% confluence according to the manufacturer’s instruction.

Oligonucleotides including controls and target-specific siRNAs were obtained from GenePharma (Shanghai, China) or Ribobio (Guangzhou, China), and the Supplementary Table S5 presents the sequences. We transfected cells with 40 nM siRNAs using Lipofectamine 2000 (Invitrogen, Grand Island, NY) at 50% confluence according to the instructions of the manufacturer. We selected one or two oligonucleotides with maximal knockdown efficiency from three different sequences to conduct the following experiments. The experiments were run in triplicate.

Lentivirus encoding c-Myc (GV358-GFP-Puro-c-Myc) and control lentivirus (GV358-GFP-Puro) were obtained from Genechem, Co., Ltd. (Shanghai. China). Lentivirus encoding NAF1 (PHBLV-GFP-Puro-NAF1) and control lentivirus (PHBLV-GFP-Puro) were purchased from HanBio Biotechnology, Co., Ltd (Shanghai, China). Lentivirus encoding NAF1 shRNA (PHBLV-GFP-Puro-sh-NAF1) and control lentivirus (PHBLV-GFP-Puro-sh-NC) were obtained from HanBio Biotechnology, Co., Ltd, and the Supplementary Table S6 presents the sequences. Cells were transfected at 50% confluence with a final lentivirus multiplicity of infection (MOI) of 1–30 according to the instructions of the manufacturer.

### Dual-luciferase reporter system

For the construction of luciferase reporter plasmids, we amplified the promoter of NAF1 gene from genomic DNA of SF295 cells and digested the amplification product with restriction enzymes. Then, the digested production was inserted into predigested pGL3-Basic luciferase vector (Promega Corp., WI) to construct the luciferase reporter plasmids pGL3-NAF1-Luc. Supplementary Table S7 presents the primers used for plasmid construct, and the construct was confirmed by Sanger sequencing. To test promoter activity of NAF1 gene modulated by c-Myc, NRF2 and TERT, SF295 cells expressing c-Myc, NRF2 and TERT, and control cells were cotransfected with pGL3-NAF1-Luc or pGL3-Basic-Luc, and pRL-TK plasmids (Promega Corp., WI, USA), which express Renilla luciferase and was used as an internal control to normalize transfection efficiency. Next, we used the dual-luciferase reporter assay system (Promega Corp., WI) to analyze the luciferase activity in accordance with the manufacturer’s instructions. The data were presented as relative luciferase activity (Firefly luciferase activity/Renilla luciferase activity). Each assay was run in triplicate.

### The chromatin immunoprecipitation (ChIP) assay

The ChIP assay was conducted to evaluate c-Myc, NRF2, and TERT binding to its targets using the Pierce™ Magnetic ChIP Kit (Pierce Biotechnology, IL, USA) in accordance with the manufacturer’s instruction. The rabbit polyclonal antibodies against c-Myc (sc-764, Santa Cruz) and against NRF2 (sc-722, Santa Cruz), and mouse polyclonal antibodies against TERT (NB-100-317, Novus) were used for the ChIP assay. We performed the detailed protocol as previously described^48^. We used the DNA fragments as templates to carry out the ChIP-qPCR assay, and normalized the data by respective 5% input. Supplementary Table S8 presents the primer sequences, and each assay was run in triplicate.

### Cell proliferation, apoptosis, cell migration, and invasion assays

Cell proliferation was evaluated by the MTT assay. Cell apoptosis was evaluated by flow cytometer. Cell migration and invasion ability were assessed by transwell chambers. The detailed protocols were performed as described previously^47^. Each experiment was run in triplicate.

### The soft agar colony-forming assay

The soft agar colony-formation assay was conducted using three-dimension cell culture. Briefly, we coated the six-well culture plate with 3 mL bottom agar (Sigma-Aldrich) mixture [DMEM, 10% (v/v) FBS, 0.6% (w/v) agar]. After the bottom agar mixture was solidified, we added 1 mL of top agar-medium mixture [DMEM, 10% (v/v) FBS, 0.3% (w/v) agar] containing 7.5 × 10^3^ cells, and incubated the cells at 37 °C for 2 weeks. Then, with a diameter ≥ 200 μm, we calculated the total number of colonies in more than 5 fields per well for a total of 15 fields in triplicate experiments.

### Electron tomography

Electron microscope observation of the ribosomes in glioma cells was described previously^29^.

### Sucrose gradient centrifugation

The sucrose gradient centrifugation was performed to measure the concentration of different ribosomal subunits. The protocol was described previously^49^.

### PAGE-silver nitrate staining

The fast silver stain kit was obtained from Beyotime Biotechnology, Co., Ltd (Shanghai, China) to detect protein absorbance in SDS-PAGE gels as previously described^50^.

### Measurement of protein synthesis

The Click-iT® AHA Alexa Fluor® 488 Protein Synthesis HCS Assay kit (Catalog no. C10289) was used to investigate the ability of protein biosynthesis in glioma cells. L-azidohomoalaninean is the amino acid analog of L-methionine, which contains an azido moiety and can be incorporated into proteins during protein biosynthesis. Briefly, 1 × 10^4^/mL cells were seeded on sterile coverslips in six-well plates. Next, we incubated cells with 50 μM of L-azidohomoalaninean in a methionine-free medium for 30 min. Cells were washed, fixed, and permeabilized. Then, we incubated cells with Click-iT® reaction cocktail at room temperature for 30 min with no light to detect the proteins modified by amino analog in the azido. As Alexa Fluor® 488 alkyne in the cocktail can click the azido moiety and DAPI may be used as control, we used the Olympus FluoView 1000 confocal microscope to detect the Alexa Fluor® 488 alkyne and the DAPI at excitation/emission wavelengths of 485/535 nm and 350/461 nm. The analysis was performed using ImageJ (NCBI, NIH) as previously described^32^. Each experiment was performed in triplicate.

### Coimmunoprecipitation (Co-IP)

After cells were at 90% confluence, protein extracts were generated by lysing cells with RIPA buffer at 4 °C for 1 h. The Co-IP assay was then performed as described previously^51^.

### Relative telomere length (RTL) measurement

The qPCR assay was used to detect the relative telomere length (RTL) as previously described^52^. Supplementary Table S9 presents the primer sequences. Each experiment was performed three times.

### Immunofluorescence assay

First, we placed cells onto a sterile sheet in a six-well plate, and treated cells with different reagents at the optimal concentration for 48 h. Then, we fixed cells with 4% paraformaldehyde at room temperature for 15 min, and used 0.3% Triton X-100 to permeabilize cells for 10 min. Next, we used goat serum to block cells for 30 min, and used primary antibodies (Supplementary Table S3) to incubate cells overnight at 4 °C. After that, goat–anti-rabbit secondary antibody was used to incubate cells at 37 °C for 90 min in the dark, and Hoechst 33342 was used for nuclear counterstaining. The confocal laser scanning microscopy (CLSM; Leica TCS SP5) was used to take the images.

### Animal studies

Three- to four-week-old male athymic nude mice were obtained from SLAC laboratory Animal Co., Ltd, and were randomly divided into four groups (five mice per group). The right armpit region of nude mice was subcutaneously inoculated of 8 × 10^6^ U87 cells stably knocking down NAF1 or 6 × 10^6^ U87 cells stably expressing NAF1 and the same number of control cells to establish tumor xenografts. Three days after injection, we measured tumor size every 2 days for 15 days, and calculated tumor volumes by the formula (length × width^2^ × 0.5). The mice were killed at the end of the study, and tumors were harvested and weighted. Xenograft tumors were then embedded in paraffin and sectioned until use. The animal studies were approved by Laboratory Animal Center of Xi’an Jiaotong University and all of the experimental procedures were performed according to Institution Guidelines.

### Statistical analysis

Gene expression in control subjects and tumor tissues was compared by the unpaired *t*-test. Survival curves were performed in accordance with the Kaplan–Meier method, and statistical analysis was conducted by the log-rank test. The association of NAF1 expression with clinicopathological or genetic characteristics is assessed by Fisher’s exact test. The correlation analysis between genes was analyzed by linear regression test. The data were shown as mean ± SD unless otherwise noted. The SPSS statistical package (11.5, Chicago, IL) was used to perform all statistical analyzes. *P* values < 0.05 were considered significantly.

## Supplementary information

Supplementary information.
