# Supplementary material for: Increased expression of NAF1 contributes to malignant phenotypes of glioma cells through promoting protein synthesis and associates with poor patient survival
Source: Oncogenesis. 2019 Apr 1;8(4):25. doi: 10.1038/s41389-019-0134-2 (PMC6443650; doi:10.1038/s41389-019-0134-2)
Supplement: Supplementary file 1 — Supplementary information. [file 41389_2019_134_MOESM1_ESM.doc]

**Supplementary information**

**Table S1.** Association of *NAF1* expression with clinicopathologic and genetic characteristics in gliomas

| **Variables** |  | ***NAF1* expression** | | *P* value |
| --- | --- | --- | --- | --- |
|  | Number | High (%) | Low (%) |  |
| **Age** |  |  |  |  |
| <47 | 298 | 144 (48) | 154 (52) | 0.256 |
| ≥47 | 298 | 153 (51) | 145 (49) |  |
| **Gender** |  |  |  |  |
| Female | 344 | 177 (51) | 167 (49) | 0.258 |
| Male | 252 | 122 (48) | 130 (52) |  |
| **Tumor grade** |  |  |  |  |
| LGGs | 514 | 236 (46) | 278 (54) | 0.005 |
| GBMs | 142 | 83 (58) | 59 (42) |  |
| **OS Status** |  |  |  |  |
| Dead | 170 | 99 (58) | 71 (42) | 0.006 |
| Alive | 426 | 198 (46) | 228 (54) |  |
| ***ATRX* status** |  |  |  |  |
| Mutant | 196 | 77 (39) | 119 (61) | 0.001 |
| WT | 460 | 242 (53) | 218 (47) |  |
| ***IDH1/2* status** |  |  |  |  |
| Mutant | 264 | 140 (53) | 124 (47) | 0.173 |
| WT | 119 | 70 (59) | 49 (41) |  |
| ***TERT* promoter status** |  |  |  |  |
| Mutant | 31 | 10 (32) | 21 (68) | 0.287 |
| WT | 23 | 10 (43) | 13 (57) |  |

**Abbreviations**: Wild-type (WT); Low Grade Gliomas (LGGs); Glioblastoma Multiformes (GBMs); Overall Survival Status (OS Status)

**Table S2**. The primer sequences used in this study for qRT-PCR assay

| **Genes** | **RefSeq ID** | **Forward primer (5′-3′)** | **Reverse primer (5′-3′)** | **Length** |
| --- | --- | --- | --- | --- |
| *NAF1* | NM_138386.2 | ATATTCGAGATATTTGGACCTGTTG | CTCTGGTGGTGGTTCCTGAT | 213bp |
| *c-Myc* | NM_002467.5 | CACCGAGTCGTAGTCGAGGT | TTTCGGGTAGTGGAAAACCA | 93bp |
| *NRF2* | NM_006164.4 | ACACGGTCCACAGCTCATC | TCTTGCCTCCAAAGTATGTCAA | 99bp |
| *TERT* | NM_198253.2 | CGTGGTTTCTGTGTGGTGTC | CCTTGTCGCCTGAGGAGTAG | 214bp |
| *SNORNA-73* | NM_002907.2 | ATACACCCGGGAGGTCACTC | TGTTTCCTGCATGGTTTGTC | 194bp |
| *5'-ETS-18S* | NM_145819.1 | GCGCCCGTCGGCATGTATTAGCTC | CTCGCCGCGCTCTACCTTACCTACCTGG | 209bp |
| *TERC* | NM_001566.1 | AACCCTAACTGAGAAGGGCG | TGACAGAGCCCAACTCTTCG | 271bp |
| *β-actin* | NM_001101.4 | GCACAGAGCCTCGCCTT | GTTGTCGACGACGAGCG | 93bp |
| *SNRNA-U6* | NM_004394.1 | CGCTTCGGCAGCACATATAC | AAAATATGGAACGCTTCACG | 101bp |

**Table S3**. The antibodies used in this study

| **Antibodies** | **Catalog#** | **Source** |
| --- | --- | --- |
| anti-NAF1 for IHC | ab122323 | (Abcam) |
| anti-NAF1 for WB | ab157106 | (Abcam) |
| anti-GAPDH | M20006 | (Abmart) |
| anti-c-Myc for WB | sc-764 | (Santa Cruz) |
| anti-c-Myc for IHC | sc-40 | (Santa Cruz) |
| anti-NRF2 | sc-722 | (Santa Cruz) |
| anti-TERT | NB-100-317 | (Novus) |
| anti-Actin | sc-1616 | (Santa Cruz) |
| anti-GFP | sc-9996 | (Santa Cruz) |
| anti-POLR1A | sc-48385 | (Santa Cruz) |
| anti-POLR2A | sc-55492 | (Santa Cruz) |
| anti-IgG | sc2025 | (Santa Cruz) |
| anti-p53 | sc126 | (Santa Cruz) |
| anti-MDM2 | sc-965 | (Santa Cruz) |
| anti-NPM1 | sc-271737 | (Santa Cruz) |
| anti-RPS14 | sc-68873 | (Santa Cruz) |
| anti-Ki67 | Cat550609 | (BD Pharmingen) |

**Abbreviations**: Immunohistochemistry (IHC); Western Blot Analysis (WB)

**Table S4.** The primer sequences used in this study for plasmid construction

| **Plasmid** | **Forward primer (5′-3′)** | **Reverse primer (5′-3′)** | **Restriction sites** |
| --- | --- | --- | --- |
| pcDNA3.1(-)A-NAF1 | AATTTGGATCCGCCACCATGGAGGTAGTGGAGGCCGCCGC | ATATAGGTACCCTAATAGTAAGGTCCAAAATGAG | *BamH* I and *Kpn* I |

**Table S5**. The sequences of siRNAs used in this study

| **siRNAs** | **Sequence (5’-3’)** | **Source** |
| --- | --- | --- |
| si-NAF1-654 (sense) | UCAGAUGGAGAUGAUGAUUTT | Gene Pharma |
| si-NAF1-654 (antisense) | AAUCAUCAUCUCCAUCUGAT | Gene Pharma |
| si-NAF1-927 (sense) | GGACCUGUUGCACAUCCAUTT | Gene Pharma |
| si-NAF1-927 (antisense) | AUGGAUGUGCAACAGGUCCTT | Gene Pharma |
| si-c-Myc (sense) | AACGATTCCTTCTAACAGA | Ribobio |
| si-c-Myc (antisense) | UCUGUUAGAAGGAAUCGTT | Ribobio |
| si-NRF2 (sense) | GCACCUUAUAUCUCGAAGUTT | Ribobio |
| si-NRF2 (antisense) | ACUUCGAGAUAUAAGGUGCTT | Ribobio |
| si-TERT (sense) | GGCCGATTGTGAACATGGA | Ribobio |
| si-TERT (antisense) | UCCAUGUUCACAAUCGGCC | Ribobio |
| si-NC (sense) | UUCUCCGAACGUGUCACGUTT | Ribobio |
| si-NC (antisense) | ACGUGACACGUUCGGAGAATT | Ribobio |

**Table S6**. The shRNAs used in this study

| **shRNAs** | **Sense (5’-3’)** | **Antisense (5’-3’)** |
| --- | --- | --- |
| sh-NAF1 | GatccGGACCTGTTGCACATCCATTTCAAGAGAATGGATGTGCAACAGGTCCTTTTTTc | aattgAAAAAAGGACCTGTTGCACATCCATTCTCTTGAAATGGATGTGCAACAGGTCCg |
| sh-NC | gatccGTTCTCCGAACGTGTCACGTAATTCAAGAGATTACGTGACACGTTCGGAGAATTTTTTc | aattcAAAAAATTCTCCGAACGTGTCACGTAATCTCTTGAATTACGTGACACGTTCGGAGAACg |

**Table S7. The primers used in** **this study for luciferase reporter plasmid construction**

| **Plasmid** | **Forward primer (5′-3′)** | **Reverse primer (5′-3′)** | **Restriction sites** |
| --- | --- | --- | --- |
| pGL3-NAF1-Luc | tctatcgataggtaccGCTGGGGATGTCCTTTACTCT | GATCGCAGATCTCGAGTCGCACCGCGCCAGAAACCGG | *Kpn* I and *Xho* I |

**Table S8.** The primers used in this study for ChIP assay

| **Gene** | **Position** | **Forward primer (5’-3’)** | **Reverse primer (5’-3’)** | **Length** |
| --- | --- | --- | --- | --- |
| NAF1 | P1: -100/-26 | GATTTTCGAAGATATGACGCATGTA | CGTGTGACGTATGTGGCG | 74bp |
|  | P2: -519/-410 | TTTGGCGCTTCAAGCAGAAT | AGGGAGGCCCTAAATCGACC | 109bp |
|  | P3: -981/-543 | ACTACACAGGGACTGATGCC | GGGGTGCCTGAAGAATGCTA | 438bp |
|  | P4: -1648/-1551 | TCTGGCTCAAACGTCTTCCC | GGAACAATGCATGAACTCACGA | 97bp |

**Table S9.** The primers used in this study for telomere length measurement

| **Genes** | **Forward primer (5’-3’)** | **Reverse primer (5’-3’)** |
| --- | --- | --- |
| *Telomere* | 5′-CGGTTTGTTTGGGTTTGGGTTTGGGTTTGGGTTTGGGTT-3′ | 5′-GGCTTGCCTTACCCTTACCCTTACCCTTACCCTTACCCT-3′ |
| *36B4* | 5′-CAGCAAGTGGGAAGGTGTAATCC-3′ | 5′-CCCATTCTATCATCAACGGGTACA A-3′ |

**Supplementary figure legends**

**Figure S1.** Linear regression analysis was performed to investigate the association of mRNA expression of NAF1 with mRNA expression of c-Myc (R =0.36; *P* <0.0001) and NRF2 (R =0.21; *P* <0.0001) in gliomas (data from TCGA dataset).

**Figure S2. The regulation of NAF1 expression by TERT.** (**a**, **b**) Upon knocking down of TERT in SF295 and U87 cells using si-TERT, the protein and mRNA expression of TERT and NAF1were measured by western blot and qRT-PCR assays. GAPDH was used as loading control in western blot analysis and the western blot is representative of three independently preformed experiments. *β-actin* mRNA was used as a normalized control for qRT-PCR assay. (**c**, **d**) Western blot and qRT-PCR assays were performed to detect protein and mRNA expression of TERT and NAF1 in SF295 and U87 cells ectopically expressing TERT and controls. GAPDH was used as a loading control in western blot analysis and the western blot is representative of three independently preformed experiments. *β-actin* mRNA was used as a normalized control for qRT-PCR assay. Data were presented as mean ± SD, * *P* < 0.05; ** *P* < 0.01 (n =3).

**Figure S3.** SF295 cells expressing TERT and control cells were subjected to ChIP-qPCR assays using TERT antibody. P1-P4 indicated four different regions of *NAF1* promoter (P1: -100/-26; P2: -519/-410; P3: -981/-543; P4: -1648/-1551) (Figure 2**k**, left panel). Fold enrichment was shown as mean ± SD, ** *P* < 0.01 (n =3).

**Figure S4.** Dual-Luciferase Reporter assay system was used to test the effect of ectopic expression of

TERT on promoter activity of NAF1 in SF295 cells. The empty vector was used as the control, and all the ratio of the Luc/Renilla activity were shown as means ± SD. ***, *P* <0.001 (n =3).

**Figure S5.** (**a**) Inhibition of mRNA expression levels of NAF1 by using two different siRNAs (si-NAF1-654 and si-NAF1-927) in SF295 and U87 cell lines were evidenced by qRT-PCR assay. *β-actin* mRNA was used as a normalized control for qRT-PCR assay. Data were presented as mean ± SD, ** *P* < 0.01 (n =3). (**b**) Ectopic expression of NAF1 were confirmed by qRT-PCR assays in SF295 and U87 cells. *β-actin* mRNA was used as a normalized control for qRT-PCR assay. Data were presented as mean ± SD, ** *P* < 0.01 (n =3).

**Figure S6. *In vitro* tumor-promoting role of NAF1 in SHG44 cells.** (**a**) Inhibition efficiency of two different siRNAs (si-NAF1-654 and si-NAF1-927) in SHG44 cells was validated by western blot analysis. Actin was used as a loading control. (**b**) Cell viability upon NAF1 knockdown in SHG44 cells was determined by the MTT assay. *, *P* <0.05; ***, *P* <0.001 (n =5). (**c**) Left panels show the representative images of colony formation in soft agar in SHG44 cells upon NAF1 knockdown. Scale bars, 50 μm. Quantitative analysis of colony numbers (right panels). **, *P* <0.01 (n =5). (**d**) Cell apoptosis was measured in SHG44 cells by flow cytometry analysis. ***, *P* <0.001 (n =3). (**e**) The left panels are representative images of migrated/invaded cells upon NAF1 knockdown. The quantitative analysis of the number of migrated/invaded cells (right panels). Scale bars, 100 μm. **, *P* <0.01; ***, *P* <0.001 (n =5). (**f**) Ectopic expression of NAF1 was confirmed in U251 cells by western blot analysis. GAPDH was used as a loading control. (**g**) Cell viability upon NAF1 overexpression in U251 cells was determined by the MTT assay. **, *P* <0.01 (n =5). (**h**) The representative images of colony formation in soft agar upon NAF1 overexpression in U251 cells (left panels). Scale bars, 50 μm. Quantitative analysis of colony numbers (right panels). **, *P* <0.01 (n =5). (**i**) The left panels are representative images of migrated/invaded cells upon NAF1 overexpression. Quantitative analysis of the number of migrated/invaded cells (right panels). Scale bars, 100 μm. Data were presented as mean ± SD. ***, *P* <0.001 (n =5).

**Figure S7.** SF295 cells were collected and subjected to western blot analysis to determine the interaction between POLR2A and NAF1 using co-immunoprecipitation with corresponding antibodies. The antibody IgG was used as negative control and the co-immunoprecipitation is representative of three independently preformed experiments.

**Figure S8.** qRT-PCR and qPCR assays were performed to measure *TERC* expression and relative length of telomere upon knockdown (**a**, **b**) and ectopic expression of NAF1 (**c**, **d**) in SF295 and U87 cells, respectively. *SNRNA-U6* was used as an endogenous control for normalizing *TERC* expression, while the single copy gene, *36B4*, was used as a reference for telomere length measurement. Data were presented as mean ± SD. **, *P* <0.01; ***, *P* <0.001 (n =3).

**Figure S9.** (**a**) Representative images of immunohistochemistry staining show the Ki67 levels of U87 cell-derived xenograft tumors from NAF1-knockdown and control groups (left panels). Scale bars, 200 μm. The right panel shows the number of Ki67-positive cells from five microscopic fields in each group. ***, *P* <0.001 (n =5). (**b**) Representative images of immunohistochemistry staining show the Ki67 levels of U87 cell-derived xenograft tumors from NAF1-overexpressing and control groups (left panels). Scale bars, 200 μm. The right panel represents the number of Ki67-positive cells from five microscopic fields in each group. Data were presented as means ± SD.***, *P* <0.001 (n =5).

**Figure S10.** Shown in the panels (**a**, **b**) are the quantitative illustration of the levels of the indicated proteins using densitometry to measure the density of the corresponding bands on the western blot shown in the figure 7**e**, **f**, respectively. **, *P* <0.01; ***, *P* <0.001 (n =3). (**c**, **d**) Bar graphs represent the number of positive cells of IHC staining shown in the figure 7**g**, **h** from five microscopic fields in each group. Data were presented as mean ± SD. ***, *P* <0.001 (n =5).

**Supplementary Fig. S1**

**
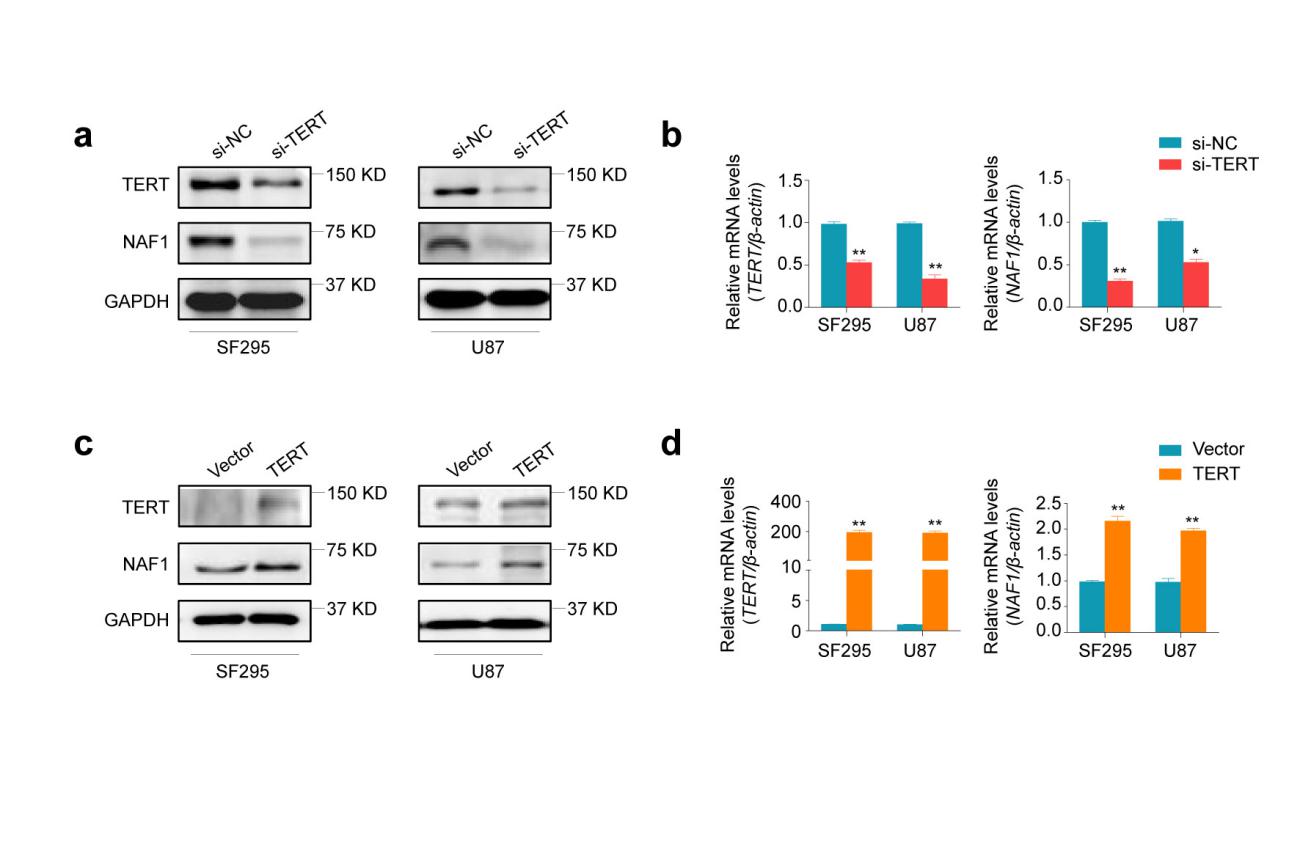
**

**Supplementary Fig. S2**

**Supplementary Fig. S3**

**Supplementary Fig. S4**


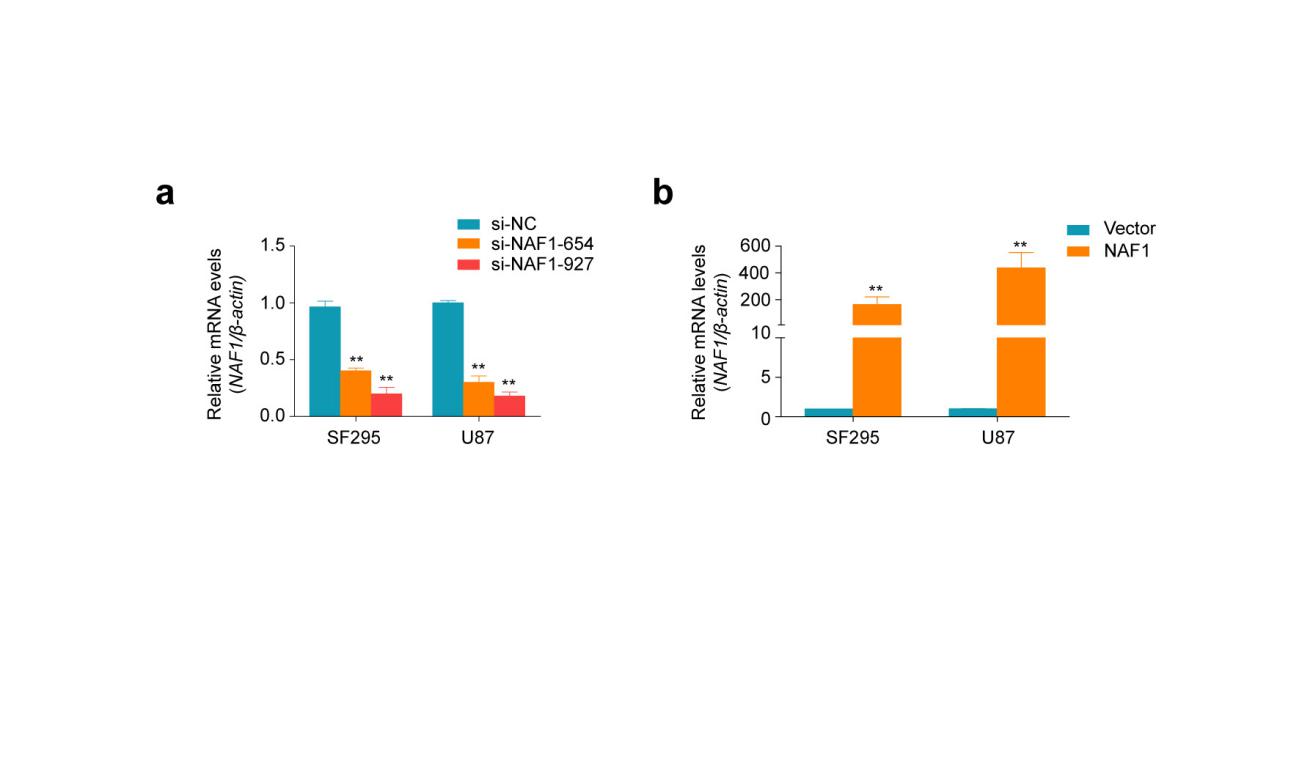


**Supplementary Fig. S5**


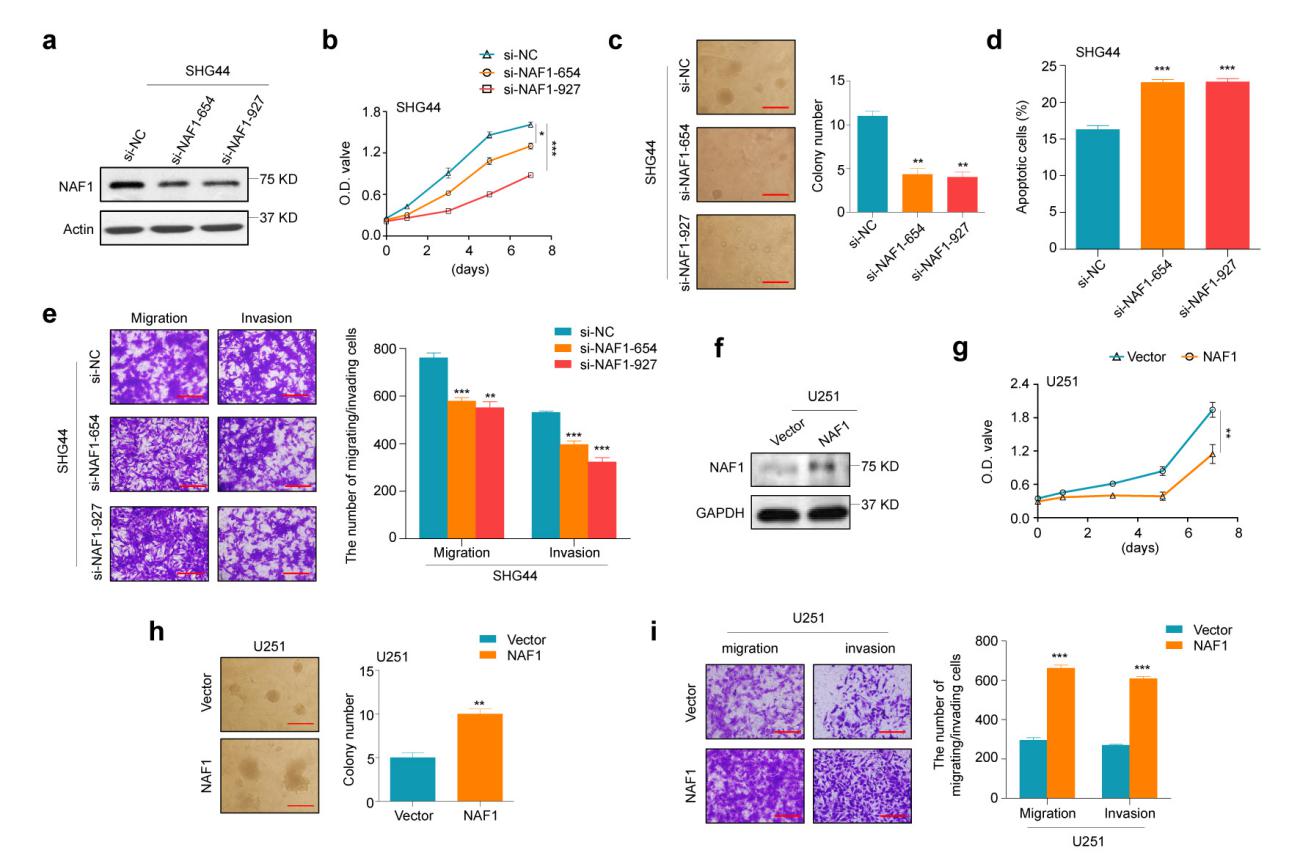


**Supplementary Fig. S6**

**Supplementary Fig. S7**

**
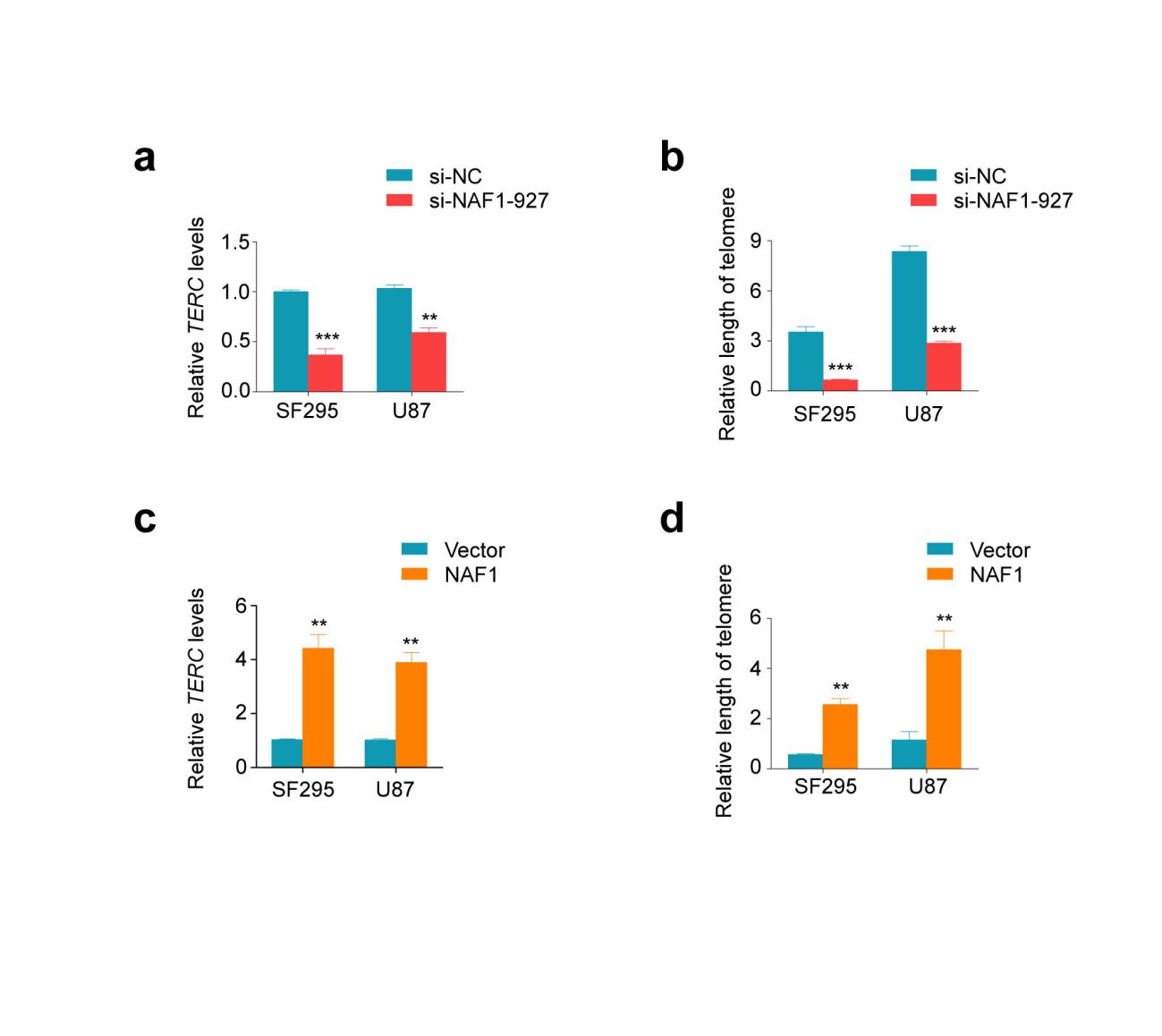
**

**Supplementary Fig. S8**

**
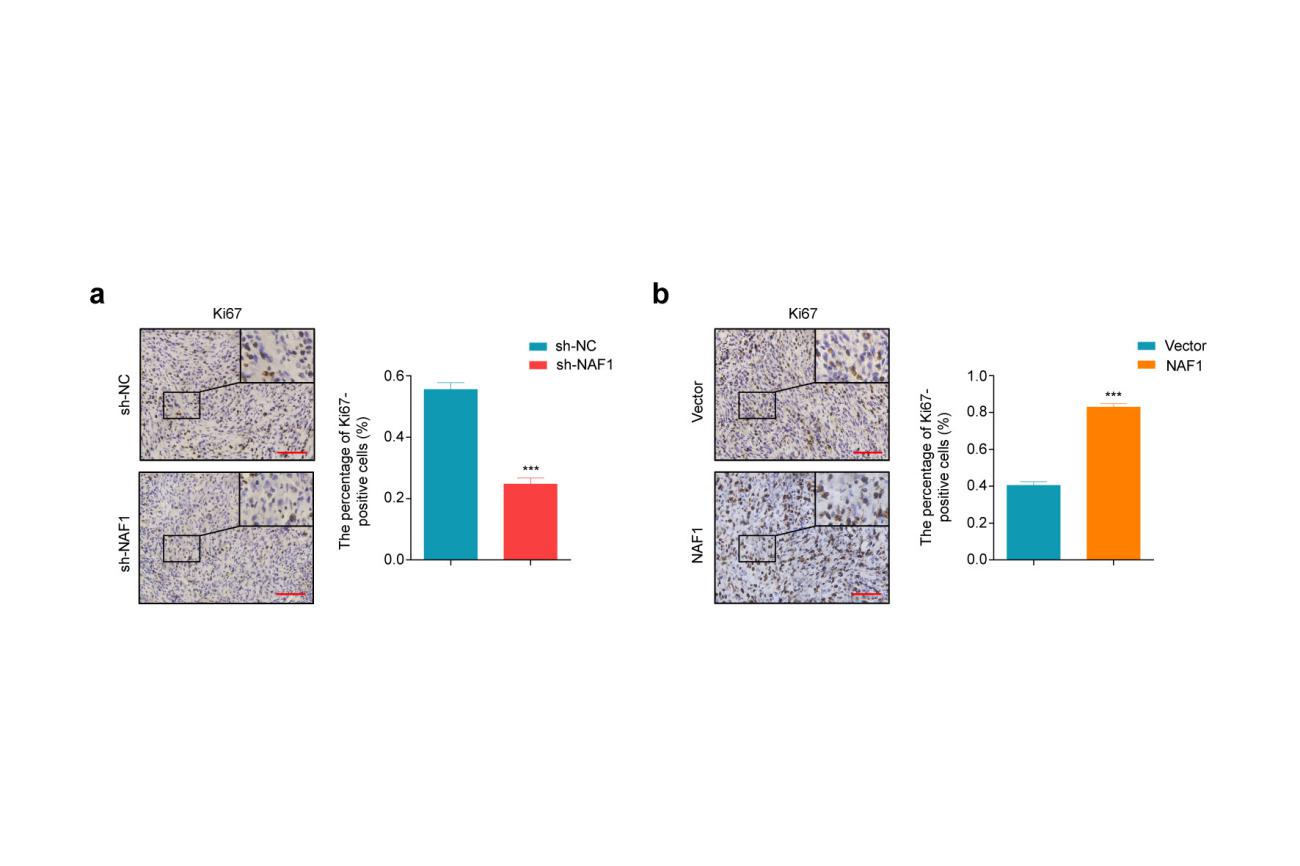
**

**Supplementary Fig. S9**

**
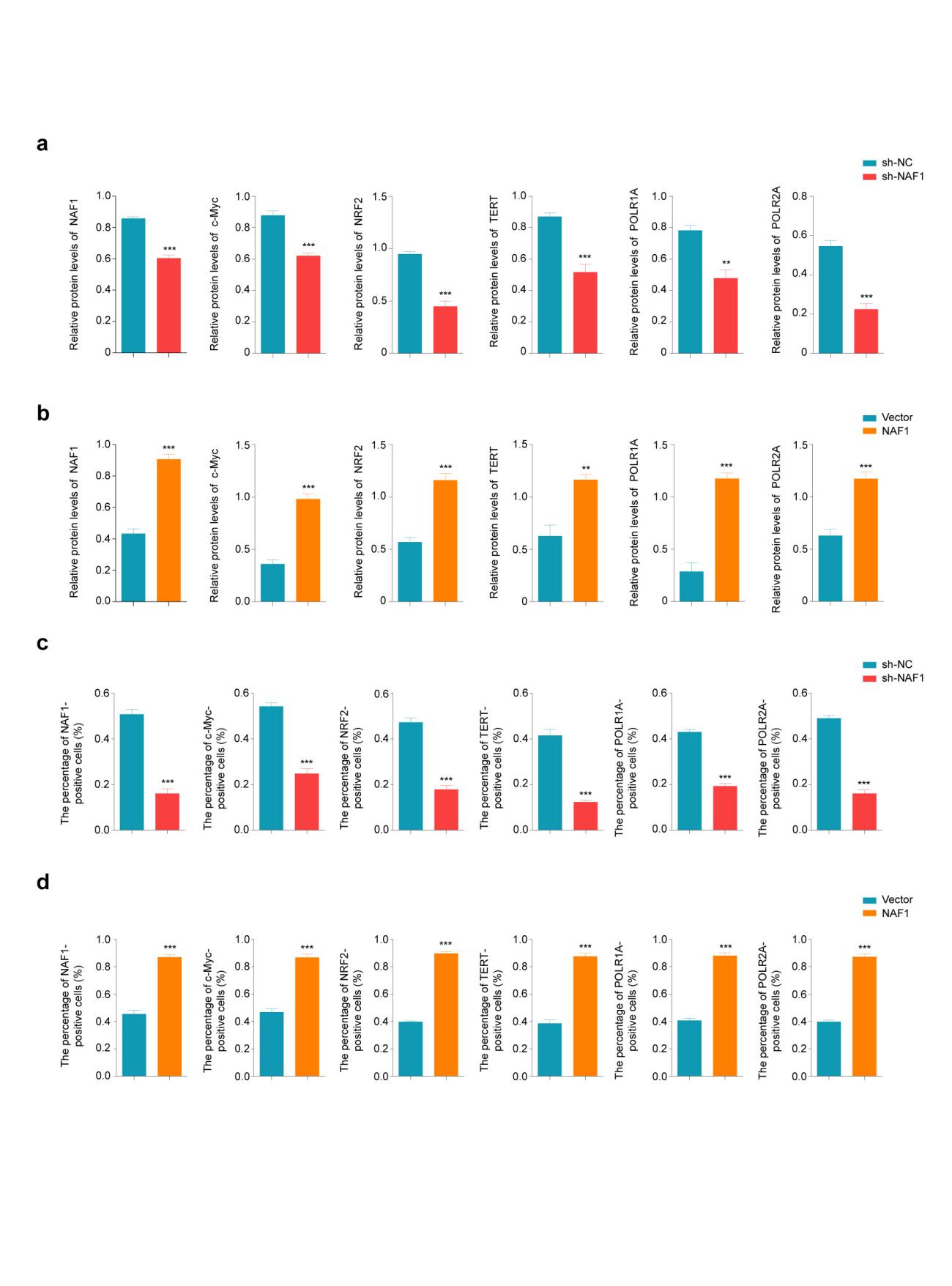
**

**Supplementary Fig. S10**
